# Expanding the *Pseudomonas* diversity of the wheat rhizosphere: four novel species antagonizing fungal phytopathogens and with plant-beneficial properties

**DOI:** 10.3389/fmicb.2024.1440341

**Published:** 2024-07-15

**Authors:** Noémie Poli, Christoph Joseph Keel, Daniel Garrido-Sanz

**Affiliations:** Department of Fundamental Microbiology, University of Lausanne, Lausanne, Switzerland

**Keywords:** *Pseudomonas*, phylogenomcis, diversity, novel species, rhizosphere, wheat, plant-beneficial bacteria, fungal phytopathogen antagonism

## Abstract

Plant-beneficial *Pseudomonas* bacteria hold the potential to be used as inoculants in agriculture to promote plant growth and health through various mechanisms. The discovery of new strains tailored to specific agricultural needs remains an open area of research. In this study, we report the isolation and characterization of four novel *Pseudomonas* species associated with the wheat rhizosphere. Comparative genomic analysis with all available *Pseudomonas* type strains revealed species-level differences, substantiated by both digital DNA-DNA hybridization and average nucleotide identity, underscoring their status as novel species. This was further validated by the phenotypic differences observed when compared to their closest relatives. Three of the novel species belong to the *P. fluorescens* species complex, with two representing a novel lineage in the *Pseudomonas* phylogeny. Functional genome annotation revealed the presence of specific features contributing to rhizosphere colonization, including flagella and components for biofilm formation. The novel species have the genetic potential to solubilize nutrients by acidifying the environment, releasing alkaline phosphatases and their metabolism of nitrogen species, indicating potential as biofertilizers. Additionally, the novel species possess traits that may facilitate direct promotion of plant growth through the modulation of the plant hormone balance, including the ACC deaminase enzyme and auxin metabolism. The presence of biosynthetic clusters for toxins such as hydrogen cyanide and non-ribosomal peptides suggests their ability to compete with other microorganisms, including plant pathogens. Direct inoculation of wheat roots significantly enhanced plant growth, with two strains doubling shoot biomass. Three of the strains effectively antagonized fungal phytopathogens (*Thielaviopsis basicola*, *Fusarium oxysporum*, and *Botrytis cinerea*), demonstrating their potential as biocontrol agents. Based on the observed genetic and phenotypic differences from closely related species, we propose the following names for the four novel species: *Pseudomonas grandcourensis* sp. nov., type strain DGS24^T^ ( = DSM 117501^T^ = CECT 31011^T^), *Pseudomonas purpurea* sp. nov., type strain DGS26^T^ ( = DSM 117502^T^ = CECT 31012^T^), *Pseudomonas helvetica* sp. nov., type strain DGS28^T^ ( = DSM 117503^T^ = CECT 31013^T^) and *Pseudomonas aestiva* sp. nov., type strain DGS32^T^ ( = DSM 117504^T^ = CECT 31014^T^).

## Introduction

*Pseudomonas* is one of the most diverse genera of *Gammaproteobacteria*, currently comprising more than 300 validly published species.^1^ This extensive species diversity is a consequence of the relatively large genomes typical of environmental bacteria, which allows them to thrive in a wide variety of environments (Spiers et al., 2000; Silby et al., 2009, 2011; Lopes et al., 2018). *Pseudomonas* bacteria exhibit versatile lifestyles. They are found both as free-living bacteria and in association with various hosts, including animals, plants, and fungi (Silby et al., 2011). Despite the pathogenic potential of certain species, notably *P. aeruginosa* in humans (Qin et al., 2022), or *P. syringae* in plants (Xin et al., 2018), a considerable number of *Pseudomonas* species establish beneficial interactions with plants. Examples include the root-associated *P. protegens*, *P. chlororaphis*, *P. brassicacearum* or *P. ogarae* (Haas and Défago, 2005; Ramette et al., 2011; Garrido-Sanz et al., 2016, 2021, 2023b), which all belong to the *Pseudomonas fluorescens* species complex (Garrido-Sanz et al., 2016). Consequently, *Pseudomonas* is widely recognized as one of the most important bacterial genera containing plant-beneficial members, making it a key target for the discovery of novel inoculants tailored to meet specific agricultural needs. This includes rhizosphere inoculants with the ability to stimulate plant growth and defenses, antagonize specific phytopathogens or insect pests, enhance the solubilization of key nutrients, and the ability to exert these activities in association with a particular host plant.

Despite numerous efforts, the effective use of bacterial rhizosphere inoculants in agriculture still faces several challenges. In particular, the inoculant must persist in the new rhizosphere environment. This means that the inoculant must compete effectively and establish itself within the native rhizosphere microbial community, in addition to being able to cope with the specific physicochemical and spatial characteristics of the soil and rhizosphere environment (Durán et al., 2021; Haskett et al., 2021; Burz et al., 2023; Garrido-Sanz et al., 2023a; Vacheron et al., 2023). Once the inoculant overcomes these challenges, its association with the plant rhizosphere can lead to beneficial outcomes. Direct plant-beneficial effects by *Pseudomonas* inoculants are primarily achieved by increasing the availability of plant growth limiting nutrients, such as phosphate or nitrogen (Bakker et al., 2018; Trivedi et al., 2020). In addition, the bacterial modulation of the plant hormonal balance can result in direct beneficial effects on the plant. For example, regulation of plant ethylene concentration by bacterial 1-aminocyclopropane-1-carboxylate (ACC) deaminase activity confers tolerance to moderate salinity and drought (Glick, 2005; Glick et al., 2007; Ilangumaran and Smith, 2017). Also, stimulation of root development by bacterial biosynthesis of auxin phytohormones can in turn enhance plant nutrient acquisition (Spaepen et al., 2007; Olanrewaju et al., 2017). Indirectly, *Pseudomonas* inoculants can have plant-beneficial effects by priming plants for defense or by inducing systemic plant resistance responses (Iavicoli et al., 2003; Weller et al., 2012; Mauch-Mani et al., 2017), or by antagonizing phytopathogens through the production of antimicrobial compounds or toxins (Haas and Défago, 2005). Specifically, the antagonistic behavior of pseudomonads against fungal and other plant pathogens relies on the release of potent broad-spectrum antimicrobial compounds, including 2,4-diacetylphloroglucinol, phenazines, cyclic lipopeptides, hydrogen cyanide or other specific toxins (Gordee and Matthews, 1969; Howell and Stipanovic, 1980; Marchand et al., 2000; Iavicoli et al., 2003; Ramette et al., 2011; Loper et al., 2016), some of which also contribute to the insecticidal abilities of certain strains (Péchy-Tarr et al., 2008; Keel, 2016; Vacheron et al., 2019; Garrido-Sanz et al., 2023b).

The description of novel bacterial species relies on a polyphasic approach that integrates both phenotypic characterization and genomic information to support the description of the proposed taxon (Vandamme et al., 1996; Prakash et al., 2007; Ramasamy et al., 2014). Traditionally, genomic validation has leaned on the DNA-DNA hybridization (DDH) ‘gold standard’ of systematics in prokaryotes, requiring a value below 70% DDH relative to the closest species to establish the status of a novel species, further supported by differences in the small ribosomal subunit (16S rRNA) gene (Prakash et al., 2007). With the emergence and wide adoption of sequencing technologies, the use of whole-genome comparative approaches is becoming largely used to infer the taxonomy of large groups of bacteria, as they provide greater resolution than single genes by using the entire genetic information of the bacterium (Ramasamy et al., 2014). These methods mimic the taxonomic 70% DDH threshold, either by using its own scale, such as 95-96% of Average Nucleotide Identity (ANI) (Richter and Rosselló-Móra, 2009), or by generating a digital version of DDH values (dDDH) using the Genome Blast Distance Phylogeny (GBDP) algorithm (Goris et al., 2007; Meier-Kolthoff et al., 2013, 2022). These genome-based methods have proven effective in resolving the taxonomy of large bacterial genera, such as *Pseudomonas*, where the division into multiple major phylogenomic groups and subgroups, together with frequent taxonomic reorganizations, adds to the complexity (Garrido-Sanz et al., 2016, 2021, 2023b; Lalucat et al., 2020, 2022; Girard et al., 2021; Mulet et al., 2024).

In this work, we report the identification and characterization of four bacteria isolated from the rhizosphere of wheat. The four isolates exhibit genetic and phenotypic characteristics that clearly distinguish them from their closest relatives and thus represent novel species of the genus *Pseudomonas*, for which we propose the names *Pseudomonas grandcourensis* sp. nov., type strain DGS24^T^, *Pseudomonas purpurea* sp. nov., type strain DGS26^T^, *Pseudomonas helvetica* sp. nov., type strain DGS28^T^, and *Pseudomonas aestiva* sp. nov., type strain DGS32^T^. Functional annotation of their genomes provides further evidence that these strains possess specific traits involved in competitive colonization of the rhizosphere environment and plant growth promotion. Direct growth promotion of wheat plants upon root inoculation, along with their antagonism to three fungal pathogens in dual culture assays, demonstrates their efficacy as inoculants to enhance both plant growth and health.

## Methods

### Strains isolation, growth, and transmission electron microscopy

The four *Pseudomonas* strains described in this study were isolated from the rhizosphere of wheat (*Triticum aestivum* cv. Arina) growing in a previously designed sterile microcosm (Garrido-Sanz et al., 2023a), containing a soil extract from a previously sampled Swiss soil [46.884947N, 6.922562E, (Harmsen et al., 2024)]. Briefly, wheat seeds were surface disinfected with 4% NaOCl solution, thoroughly rinsed with sterile water and germinated on 1% agar plates as previously described (Garrido-Sanz et al., 2023a). The germinated seeds were then transferred to the microcosms. The soil wash was prepared by mixing the soil with mineral medium (MM, 1:1) (Garrido-Sanz et al., 2023a) for 1 h at 180 rpm, followed by low speed centrifugation at 300 x *g* for 1 min to remove soil debris. The supernatant was then centrifuged at 4,500 x *g* for 15 min and the pellet was washed twice with MM. This suspension contained the microbial community of the original soil and was added to the microcosms to make up 10% (w/v). Plants were grown in this system for seven days in a Percival PGC-7L2 growth chamber maintaining 70% relative humidity and a 16/8 h, 22/18 C light/dark (160 μE m^–2^ S^–1^) photoperiod. The roots with adhering soil from the wheat plants that developed were pooled by four and mixed with MM (1:1, w/v). Samples were then vortexed for 20 min to detach bacteria from the root surfaces and soil particles, and centrifuged at 300 x *g* for 1 min. The supernatant was serially diluted in MM, plated on R2A medium (Oxoid) and incubated at 25°C for 48 h. Individual colonies were picked and repeatedly streaked on R2A plates until pure cultures were obtained. Bacteria were cryopreserved at −80°C in 50% (v/v) glycerol:MM solution. Strains were routinely grown on R2A agar or nutrient agar (NA; Oxoid), or in nutrient yeast broth (NYB, per L of H_2_0: 25 g of Nutrient Broth No. 2 (Oxoid) and 5 g yeast extract (Oxoid)) at 25°C. The four novel *Pseudomonas* described in this study are part of a larger screening process to characterize wheat-associated bacteria in a naturally disease-suppressive field soil (Harmsen et al., 2024).

To prepare samples for transmission electron microscopy, cells of the four strains were grown on NYB to an optical density of 0.8 at 600 nm (OD_600_). Three μL of bacterial cultures were placed on a 400 mesh copper carbon film grid (CF400-Cu, EMS, Hatfield, PA) for 1 min. The grids were washed twice with a drop of H_2_O and stained with 1% uranyl acetate (Sigma, St Louis, MO, US) for 1 min. Finally, excess water was removed from the grids with absorbent paper. Micrographs were taken on a transmission electron microscope JEOL JEM-2100Plus (JEOL Ltd., Akishima, Tokyo, Japan) at an acceleration voltage of 80 kV with a TVIPS TemCamXF416 digital camera (TVIPS GmbH, Gauting, Germany) using the EM-MENU v4.0 software (TVIPS GmbH, Gauting, Germany).

### Complete genome sequencing and annotation

Total DNA was extracted from 8 mL overnight cultures grown in NYB at 25°C, using the DNeasy PowerSoil Pro kit (Qiagen), according to the manufacturer’s instructions. DNA concentration was measured using the Qubit dsDNA HS assay kit (Invitrogen). Samples were stored at −20°C until sequencing. Whole genome sequencing was performed using Pacific Biosciences (PacBio) single-molecule real-time (SMRT) sequencing using Circular Consensus Sequencing (CSS) technology in a PacBio Sequel II system. Samples were sequenced at the Lausanne Genomic Technologies Facility (Lausanne, Switzerland), and *de novo* assembled and analyzed using PacBio SMRT Link v11.0 software. Genomes were annotated using prokka v1.14.6 (Seemann, 2014) and quality checked using CheckM v1.2.2 (Parks et al., 2015). Functional annotation of the four genomes was performed using EggNOG mapper v2.1.5 (Huerta-Cepas et al., 2017), based on eggNOG v.5.0 orthology data (Huerta-Cepas et al., 2019). Secondary metabolite biosynthetic gene clusters (BGCs) were predicted using AntiSMASH v7.0 (Blin et al., 2023) with default settings of KnownClusterBlast, MIBiG v3.1 (Terlouw et al., 2023) cluster, Pfam (Mistry et al., 2021) cluster, ClusterBlast, ActiveSiteFinder, Pfam-based Go term annotation, SubClusterBlast, RREFinder, and TIGRFam (Haft et al., 2013) and TFBS analysis.

### Core-genome phylogeny of the genus *Pseudomonas*

The genomes of the four strains described in this work were analyzed together with 303 genomes of *Pseudomonas* type strains (listed in Supplementary Table 1), downloaded from the NCBI RefSeq database in November 2023. OrthoFinder v2.5.5 (Emms and Kelly, 2019) was applied to amino acid sequences to identify orthologous single-copy protein sequences present in all genomes. Nine hundred and twelve protein sequences were identified and aligned using MAFFT v7.520 (Katoh and Standley, 2013) with default options. Alignments were trimmed to remove spurious sequences and poorly aligned regions using trimAl v1.2 (Capella-Gutiérrez et al., 2009) with the *-automated1* option, and then concatenated. IQ-TREE v2.2.6 (Minh et al., 2020) was used to construct a maximum-likelihood phylogeny, applying the best-fitting nucleotide substitution model through automatic model detection and ultrafast bootstrapping with up to 1,000 replicates. *Cellvibrio japonicus* strain Ueda107 was used as the outgroup.

### Digital DNA-DNA hybridization and average nucleotide identity calculation

Genomic comparisons between the four strains described in this study and 303 *Pseudomonas* type strains were performed using the Genome Blast Distance Phylogeny (GBDP) algorithm (Meier-Kolthoff et al., 2013) via the Genome-to-Genome Distance Calculator (GGDC) v3.0 web service at https://ggdc.dsmz.de/ (Meier-Kolthoff et al., 2022) to obtain digital DNA-DNA hybridization (dDDH) values and intergenomic distances using the formula 2. Average Nucleotide Identity (ANI) values were obtained using FastANI v1.33 (Jain et al., 2018). The results of the four strains described in this study were further corroborated using the Type(Strain) Genome Server [TYGS, (Meier-Kolthoff et al., 2022)] in May, 2024. Comparison between the dDDH and ANI data matrices was performed using the Mantel test within the R package vegan v2.5-7 (Dixon, 2003), using Spearman correlations and 999 permutations.

To identify genomes of strains belonging to the novel species described in this study, FastANI was used on the full set of *Pseudomonas* downloaded from the NCBI RefSeq database in June 2024, consisting of 16,762 genomes. Comparisons that achieved ANI values ≥ 95% were further used to also perform GBDP. Genomes were considered to be different strains of the same species when they also shared ≥ 70% dDDH and differed by less than 1% in GC% content.

### Phenotypic characterization

The four *Pseudomonas* strains together with the type strains of the closest species were phenotypically characterized using API 20 NE, API 50 CHB/E (bioMérieux) and Biolog Gen III Microplates (Biolog). *Pseudomonas jessenii* DSM 17150^T^ (Verhille et al., 1999), *P. laurylsulfatiphila* DSM 105097^T^ (Furmanczyk et al., 2018b), *P. laurylsulfativorans* DSM 105098^T^ (Furmanczyk et al., 2018a), *P. lini* DSM 16768^T^ (Delorme et al., 2002), *P. psychrotolerans* DSM 15758^T^ (Hauser et al., 2004), and *P. oryzihabitans* DSM 6835^T^ (Kodama et al., 1985) were obtained from the German Collection of Microorganisms and Cell Cultures (DSMZ). *P. farris* LMG 32054^T^ (Girard et al., 2021) and *P. kielensis* LMG 31954^T^ (Gieschler et al., 2021) were obtained from the Belgian Coordinated Collections of Microorganisms (BCCM). API tests were performed according to the manufacturer’s instructions using colonies grown overnight at 25°C on R2A. Results were reported after 48 h of incubation at 25°C. For Biolog GEN III tests, the wells of the plates were inoculated with 100 μL of cell suspensions in MM adjusted at an OD_600_ of 0.05 and cultures were incubated for 24 h at 25°C on a rotary shaker (700 rpm). Bacterial cell growth was measured at an OD_600_ to evaluate the ability of cells to assimilate different carbon sources or their sensitivity to different compounds. Positive results for assimilation of carbon compounds were considered when cell growth was at least twice that of the negative control, and weak results were considered when cell growth was at least 1.5 times that of the negative control. For sensitivity assays, positive results (sensitive to the compound) were considered when cell growth was less than 0.5 times that of the positive control. Values between 0.5 and 0.75 times the positive control were considered weak, and values greater than 0.75 times the positive control were considered not sensitive. Differences between phenotypic traits among the type strains tested were plotted using the R package ComplexHeatmap v2.4.3 (Gu et al., 2016). A non-metric multidimensional scaling (NMDS) analysis was performed using the vegan R package, as previously described (Garrido-Sanz et al., 2021).

### Growth promotion of wheat plants

The four strains were tested *in planta* to evaluate their ability to promote the growth of wheat (*Triticum aestivum* cv. Arina). Wheat seeds were surface disinfected by rinsing them in 4% NaClO for 15 min, followed by several washes with sterile distilled water. The seeds were germinated on 1% agar plates for two days. Plastic pots of 9 cm (⌀ top), 6 cm (⌀ bottom), and 6.5 cm (height) were filled with 150 mL of autoclaved perlite (Growslab, Switzerland). Three wheat seedlings were planted in each pot. Four pots were set up for each of the four strains tested (total of 12 replicates). The following day, the plants were inoculated with 1 mL of a cell suspension containing ∼3⋅10^6^ bacteria in sterile distilled water. The pots were regularly watered with Wuxal universal plant fertilizer solution (Hauert, Switzerland) containing per L: 57 mg nitrate (NO_3_^–^), 91.6 mg ammonium (NH_4_^++^), 49.4 mg urea (CO(NH_2_)_2_), 198 mg soluble phosphate (P_2_O_5_), 148 mg soluble potassium (K_2_O), 0.248 mg soluble B, 0.1 mg soluble Cu, 0.992 mg soluble Fe, 0.744 mg soluble Mg, 0.024 mg soluble Mo, and 0.1 mg soluble Zn. After 21 days, plants were carefully removed from the pots, and shoots and roots were separated. The length and fresh weight of the shoots were measured. The roots were then washed from the attached perlite and the fresh weight was measured. The dry weight of roots and shoots was measured after drying the samples at 30°C for one month. Significant differences between groups (*P* value ≤ 0.05) were assessed using the Kruskal-Wallis rank sum test within the agricolae v1.4–5 R package, using the Fisher’s least significant difference post hoc test, and *P* values were corrected using the false discovery rate.

### Inhibition of fungal plant pathogens

The four strains described in this study were tested for antagonistic activity against three fungal plant pathogens: *Thielaviopsis basicola* isolate ETH D127, *Fusarium oxysporum* f. sp. *radicis-lycopersici* (*Fusarium oxysporum* hereafter) isolate 22, and *Botrytis cinerea* isolate ETH D110 (Keel et al., 1992) using a method adapted from De Vrieze et al. (De Vrieze et al., 2019). Briefly, *T. basicola* and *F. graminearum* were grown on potato dextrose agar (PDA, BD Difco) for 10 and 3 days, respectively, and *B. cinerea* on malt agar (MA, Oxoid) for 7 days. Eight-mm mycelial plugs were cut from the grown cultures with a sterile cork borer and placed upside down in the center of fresh malt agar plates. Bacteria were grown overnight in NYB, as described above. Bacterial cells were centrifuged at 5,000 x *g* for 2 min, washed three times with sterile distilled H_2_O, resuspended in sterile H_2_O, and finally adjusted to an OD_600_ of 0.1. Ten μL of the bacterial suspension was applied to the plate in three equidistant spots, 15 mm from the edge of the Petri dish. Negative controls were performed by inoculating 10 μL of sterile H_2_O. Plates were placed in the dark at room temperature for 2 days for *B. cinerea*, 5 days for *F. oxysporum*, and 9 days for *T. basicola*. Four replicates were performed per condition, and two independent experimental runs were conducted. The ability of the strains to inhibit the growth of the pathogens was evaluated by comparing the mycelial area to that of the controls. Photos of each plate were taken and analyzed using ImageJ v1.53t (Schneider et al., 2012) to quantify the area of mycelial growth. The percentage of inhibition area (*Ai*) was calculated using the formula: *Ai* (%) = (*Ac*−*At*) / *Ac* 100, where *Ac* is the mean pathogen area in control plates, and *At* is the pathogen area in test plates (inoculated with bacteria). Significant differences between groups were assessed using the Kruskal-Wallis rank sum test as described above.

## Results and discussion

### General genome description and cell morphology

The complete genome sequences of the four isolated strains DGS24, DGS26, DGS28 and DGS32 were obtained by PacBio sequencing. The characteristics of genomes are summarized in Table 1. Each of the four genomes consists of a single circular chromosome, with sizes ranging from 4.98 Mbp in strain DGS32 to 6.76 Mbp in strain DGS24. No plasmids were identified. The GC% varies from 58.8% in DGS28 to 65.9% in DGS32. These values are similar to the genomes of other pseudomonads (Loper et al., 2012; Garrido-Sanz et al., 2016, 2017, 2021; Lopes et al., 2018). The average genome coverage is > 70-fold in all cases. The number of genes ranges from 4,589 in DGS32 to 6,062 in DGS24, consistent with their respective genome sizes. Ribosomal gene copies were identified in all genomes, ranging from 5 copies in DGS32 to 7 copies in DGS24 and DGS26, and at least 38 unique transfer RNAs (tRNAs) were identified in all genomes.

**TABLE 1:** Genome characteristics of the four *Pseudomonas* strains sequenced in this study.

Strain	Genus^a^	Chromosome length (bp)	Cover.	GC%	Genes	CDSs	16S rRNA copies	tRNAs/ unique
DGS24	*Pseudomonas*	6,763,687	109x	59.7	6,062	5,967	7	73/39
DGS26	*Pseudomonas*	5,931,563	122x	59.9	5,485	5,388	7	75/38
DGS28	*Pseudomonas*	6,437,446	71x	58.8	5,945	5.857	6	69/38
DGS32	*Pseudomonas*	4,982,159	112x	65.9	4,589	4,500	5	74/40

^a^Taxonomic assignment at the genus level based on CheckM statistics, 16S rRNA and whole-genome TYGS. All genomes consisted of a single circular chromosome.

**FIGURE 1:**
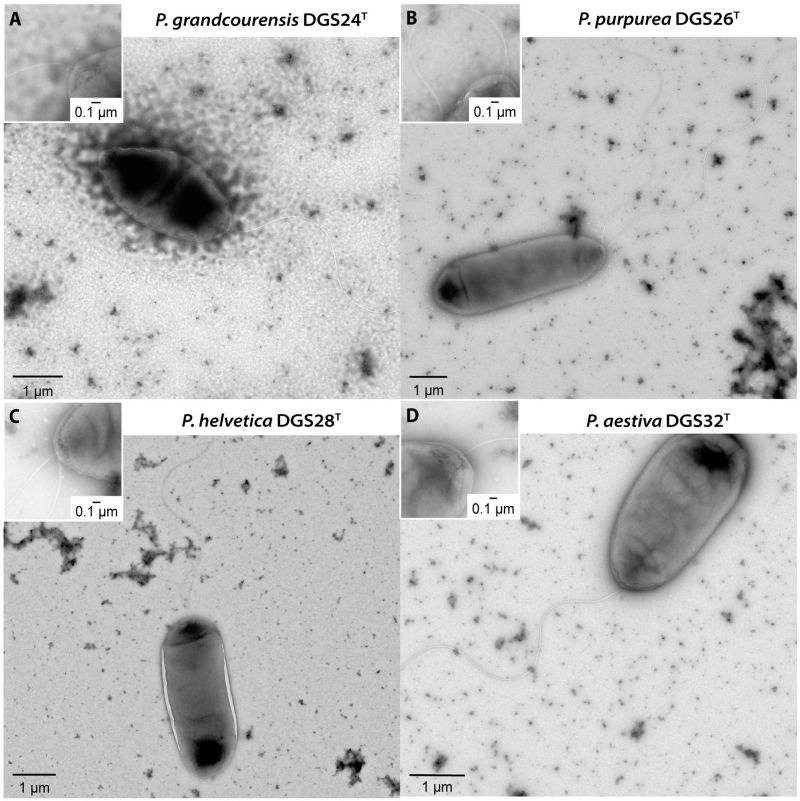
Transmission electron microscopy of cells belonging to the four *Pseudomonas* described in this study. **(A)** *Pseudomonas grandcourensis* DGS24^T^, **(B)** *Pseudomonas purpurea* DGS26^T^, **(C)** *Pseudomonas helvetica* DGS28^T^, **(D)** *Pseudomonas aestiva* DGS32^T^. Zoom-ins of the polar flagella are shown in the upper left corner of the panels.

The cells of the four isolated strains appeared rod-shaped under transmission electron microscopy (Figure 1), with cell sizes ranging from 1.5 to 1.6 μm wide and 2.9 to 3.4 μm long. Polar flagella were observed in all four strains. A single flagellar structure was observed in strains DGS24 and DGS32, while DGS26 and DGS28 had two flagella encoded by a single set of flagellar genes (see below). This observation is consistent with previous descriptions of pseudomonads (Barahona et al., 2016; Bouteiller et al., 2020).

### Genome-based phylogeny of *Pseudomonas* type strains places them in distant clades

**FIGURE 2:**
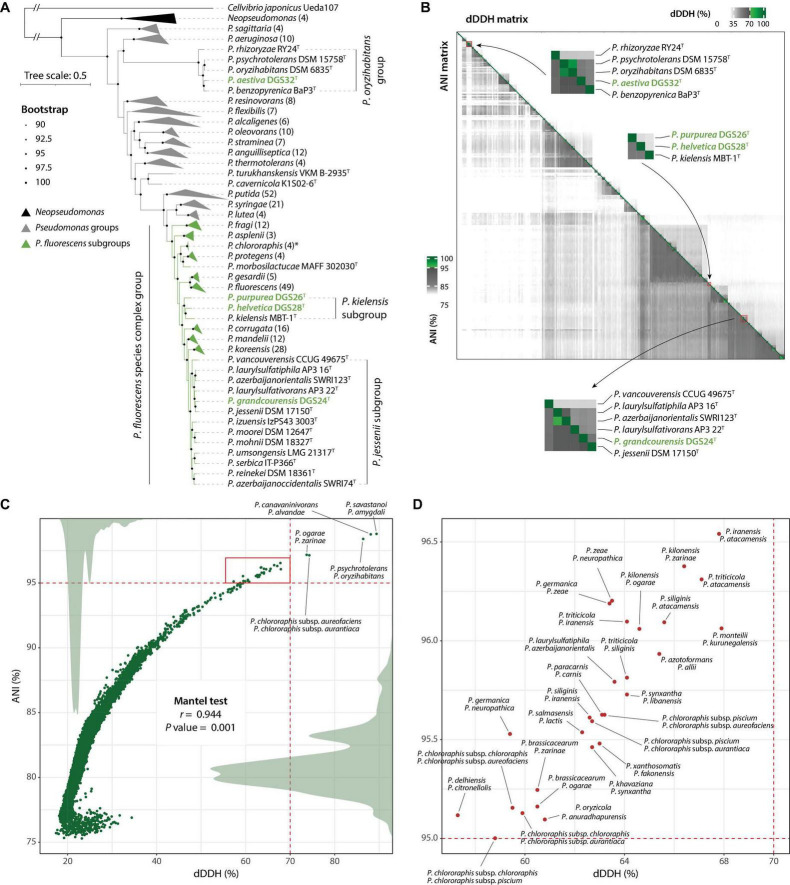
Genome-based taxonomy of *Pseudomonas* type strains. **(A)** Maximum-likelihood phylogeny of *Pseudomonas* type strains based on 912 single-copy orthologous sequences. Strains belonging to the major *Pseudomonas* groups and *P. fluorescens* subgroups are collapsed and represented as gray or green triangles, indicating the names of the groups/subgroups. The number of genomes per collapsed node is given in parentheses. Groups/subgroups containing the four strains characterized in this study (green names) have not been collapsed. An extended version of the phylogeny is provided in Supplementary Figure 1. **(B)** Digital DNA-DNA hybridization (dDDH; upper right) and Average Nucleotide Identity (ANI; lower left) data matrices. The closest comparisons to the four *Pseudomonas* species described in this work are zoomed in and labeled. Green colors in the scales indicate values above the threshold for species delineation (dDDH ≥ 70%, ANI ≥ 95%). **(C)** Correlation between dDDH and ANI matrices (Mantel test; Spearman correlation with 999 permutations). Dots indicate paired comparisons. Labeled dots indicate type strains belonging to the same species under both dDDH and ANI. Density plots of dDDH and ANI are shown as gray areas. The red square indicates discordance between dDDH and ANIb and is annotated in **(D)**.

The genomes of the four strains described in this study, together with those of 303 *Pseudomonas* type strains, were used to construct a maximum-likelihood phylogeny based on 912 single-copy orthologous sequences. The resulting phylogeny (Figure 2A, Supplementary Figure 1) shows a clear separation of the major *Pseudomonas* groups previously reported (Garrido-Sanz et al., 2016, 2021, 2023b; Lalucat et al., 2020, 2022; Girard et al., 2021). In particular, the four strains characterized in this study are found in two distant clades: *P. aestiva* DGS32 is situated within the *Pseudomonas oryzihabitans* group, representing one of the earliest diverging lineages within the genus, whereas *P. grandcourensis* DGS24, *P. purpurea* DGS26 and *P. helvetica* DGS28 are placed across the *Pseudomonas fluorescens* species complex (Figure 2A), representing the most terminal clade of the *Pseudomonas* genus. In addition to members of the *P. fluorescens* species complex, which have been largely described as plant-beneficial bacteria (Garrido-Sanz et al., 2016), species of the *P. oryzihabitans* group have also been isolated from plant roots, including type strains of *P. oryzihabitans* and *P. rhizoryzae* isolated from rice (Kodama et al., 1985; Wang et al., 2020).

We identified 11 subgroups in the *P. fluorescens* phylogenomic group. All but one have been described previously (Garrido-Sanz et al., 2016, 2023b; Girard et al., 2021). The novel group identified in this study includes three species; *P. kielensis* MBT-1^T^ and two of the species described in this work: *P. purpurea* DGS26^T^ and *P. helvetica* DGS28^T^. The three strains shared < 0.1429 of intergenomic distances (29.9-31.4% of dDDH, Supplementary Tables 2, 3), which is below the thresholds previously reported for the delineation of *P. fluorescens* subgroups (Garrido-Sanz et al., 2016, 2021, 2023b) and within the range of other subgroups within the *P. fluorescens* species complex (Supplementary Table 3). Furthermore, the closest intergenomic distance achieved outside of these three strains is 0.1455 (29.1% dDDH) with *P. lini*, which belongs to the *P. mandelii* subgroup, which is clearly a separate phylogenetic clade (Figure 2A). These results confirm the identity of a novel subgroup within the *P. fluorescens* species complex. Based on the oldest species description (Gieschler et al., 2021), we propose the name *P. kielensis* for the subgroup.

### Genome analysis confirms four novel *Pseudomonas* species and reveals taxonomic challenges

Further genome comparisons based on dDDH% and ANI of the four strains described in this study with 303 *Pseudomonas* type strains substantiated their identity as novel species (Figures 2B–D, Supplementary Table 3). None of the genome comparisons of the four novel strains achieved values above the same-species thresholds ( ≥ 70% dDDH, ≥ 95% of ANI, Table 2). The highest dDDH% value obtained was 54.3% (94.1% ANI) for *P. grandcourensis* DGS24^T^ compared to *P. jessenii* DSM 17150^T^.

**TABLE 2:** Closest *Pseudomonas* type strains to the four strains described in this study.

Strain	Closest type strain	dDDH (%)	GC% diff.	ANI (%)
*P. grandcourensis* DGS24	*P. jessenii* DSM 17150	54.3	0.0	94.1
	*P. laurylsulfativorans* AP3 22	47.1	0.08	92.58
	*P. laurylsulfatiphila* AP3 16	46.9	0.45	92.47
	*P. azerbaijanorientalis* SWRI123	46.7	0.41	92.44
	*P. azerbaijanoccidentalis* SWRI74	33.7	0.4	87.97
	*P. reinekei* DSM 18361	33.2	0.57	87.84
	*P. izuensis* IzPS43 3003	33.0	0.08	87.53
	*P. serbica* IT-P366	33.0	0.15	87.63
	*P. vancouverensis* CCUG 49675	32.9	0.08	87.9
	*P. moorei* DSM 12647	32.7	0.04	87.66
*P. purpurea* DGS26	*P. helvetica* DGS28	31.4	1.08	87.0
	*P. kielensis* MBT-1	29.9	0.98	86.17
	*P. lini* DSM 16768	29.1	1.08	85.91
	*P. frederiksbergensis* LMG 19851	29.0	0.93	85.83
	*P. silesiensis* A3	29.0	0.28	85.51
	*P. migulae* CFML 95-321	28.9	0.3	85.82
	*P. farris* SWRI79	28.8	1.12	85.9
	*P. mohnii* DSM 18327	28.6	0.24	85.37
	*P. arsenicoxydans* CECT 7543	28.5	1.11	85.74
	*P. laurylsulfatiphila* AP3 16	28.5	0.28	85.5
*P. helvetica* DGS28	*P. purpurea* DGS26	31.4	1.08	87.0
	*P. kielensis* MBT-1	30.4	2.06	85.96
	*P. frederiksbergensis* LMG 1985	29.4	0.15	85.68
	*P. lini* DSM 16768	29.4	0.0	85.92
	*P. farris* SWRI79	29.3	0.04	85.87
	*P. migulae* CFML 95-321	29.1	0.78	85.79
	*P. silesiensis* A3	29.1	0.8	85.25
	*P. nunensis* In5	28.6	0.6	85.54
	*P. arsenicoxydans* CECT 7543	28.5	0.03	85.3
	*P. azerbaijanorientalis* SWRI123	28.5	1.32	85.32
*P. aestiva* DGS32	*P. oryzihabitans* DSM 6835	52.2	0.31	93.85
	*P. psychrotolerans* DSM 15758	52.0	0.36	93.71
	*P. benzopyrenica* BaP3	49.4	0.62	93.23
	*P. rhizoryzae* RY24	35.1	1.09	89.2
	*P. delhiensis* RLD-1	22.1	2.15	80.91
	*P. citronellolis* DSM 50332	22.0	1.63	81.12
	*P. sagittaria* JCM 18195	22.0	0.74	80.79
	*P. lalucatii* R1b54	21.9	1.08	81.06
	*P. linyingensis* LMG 25967	21.9	0.47	80.81
	*P. oryzagri* MAHUQ-58	21.9	1.50	80.60

Only the top ten closest type strains are shown. For additional details see Supplementary Table 2. Digital DNA-DNA hybridization values (dDDH) and G+C% difference, were obtained using the GGDC v3 online service (https://ggdc.dsmz.de). Average Nucleotide Identity (ANI) values were calculated using FastANI.

Interestingly, our results show four cases of two strains described as different species, which in fact belong to the same species according to both dDDH and ANI (Figure 2C). These are *P. savastanoi* ICMP 4352^T^ and *P. amygdali* ICMP 3918^T^ (dDDH = 89.4%, ANI = 98.79%), *P. canavaninivorans* HB002^T^ and *P. alvandae* SWRI17 (dDDH = 88.1%, ANI = 98.75%), *P. psychrotolerans* DSM 15758^T^ and *P. oryzihabitans* DSM 6835^T^ (dDDH = 86.4%, ANI = 98.4%), and *P. ogarae* F113^T^ and *P. zarinae* SWRI108^T^ (dDDH = 73.7%, ANI = 97.18%). The other detected case of two strains belonging to the same species corresponds to the type subspecies *P. chlororaphis* subsp. *aureofaciens* and *P. chlororaphis* subsp. *aurantiaca*, indeed belonging to the same species. Recently, it has been proposed that *P. savastanoi* is a heterotypic synonym of *P. amygdali* (Todai et al., 2022). On the basis of the oldest species description, we propose an amendment to the taxonomy of *P. alvandae*, *P. oryzihabitans* and *P. ogarae* to include the most recently described species as heterotypic synonyms (see below).

Although there was a strong overall agreement between dDDH and ANI (*r* = 0.944, *P* value = 0.001), we observed differences above the species threshold in 28 cases (Figures 2B, C). In these cases, dDDH values clearly indicated different species with values below 68%, while ANI values were above 95% (above 97.5% in three comparisons), failing to distinguish between them. Many of these cases involved very closely related strains, such as the type strains of *P. ogarae*, *P. kilonensis* and *P. brassicacearum* species. The taxonomy of these species has only recently been clarified and validated as distinct species using both phenotypic and in-depth whole-genome analyses (Vacheron et al., 2018; Garrido-Sanz et al., 2021; Girard et al., 2021). Similar complex cases are likely to occur when comparing closely related species. We caution against relying solely on a single method to assess genome agreement for taxonomic purposes (Figure 2B).

In addition, we identified several *Pseudomonas* strains belonging to two of the four species described in this study, using both dDDH and ANI (Table 3). These include 22 strains belonging to *P. purpurea* and 10 strains belonging to *P. aestiva*.

**TABLE 3:** Identification of strains belonging to the novel *Pseudomonas* species.

Reference genome	Subject genome	NCBI acc. no	dDDH (%)	GC% diff.	ANI (%)
*P. purpurea* DGS26	*Pseudomonas* sp. GW456-11-11-14-LB1	GCF_002883875.1	85.6	0.07	98.41
	*Pseudomonas* sp. FW306-2-11AD	GCF_017350575.1	85.5	0.06	98.41
	*Pseudomonas* sp. FW306-02-H05-AB	GCF_017350695.1	85.6	0.05	98.41
	*Pseudomonas* sp. FW300-N2E3	GCF_001307155.1	85.4	0.02	98.40
	*Pseudomonas* sp. FW306-02-F02-AA	GCF_017350835.1	85.5	0.04	98.40
	*Pseudomonas* sp. FW306-02-H06C	GCF_017350635.1	85.5	0.03	98.40
	*Pseudomonas* sp. FW306-02-F08-AA	GCF_017350755.1	85.5	0.05	98.39
	*Pseudomonas* sp. FW306-2-11BA	GCF_017350595.1	85.5	0.05	98.38
	*Pseudomonas* sp. FW306-02-H06B	GCF_017350675.1	85.5	0.05	98.38
	*Pseudomonas* sp. FW306-2-11AA	GCF_017350655.1	85.5	0.06	98.38
	*Pseudomonas* sp. FW306-02-F04-BA	GCF_017350745.1	85.5	0.05	98.37
	*Pseudomonas* sp. FW306-02-H05-AA	GCF_017350735.1	85.5	0.06	98.37
	*Pseudomonas* sp. FW300-N1A5	GCF_017351755.1	85.5	0.04	98.37
	*Pseudomonas fluorescens* FW300-N2E3	GCF_017351665.1	85.3	0.02	98.37
	*Pseudomonas* sp. FW306-02-H05-BA	GCF_017350705.1	85.5	0.05	98.36
	*Pseudomonas* sp. FW306-2-11AB	GCF_017350615.1	85.5	0.06	98.36
	*Pseudomonas* sp. FW306-02-F02-AB	GCF_017350815.1	85.7	0.05	98.35
	*Pseudomonas* sp. FW306-02-F04-AA	GCF_017350785.1	85.5	0.05	98.35
	*Pseudomonas* sp. FW306-2-11AC	GCF_017350555.1	85.4	0.06	98.32
	*Pseudomonas* sp. GW460-R15	GCF_002901575.1	81.2	0.18	98.01
	*Pseudomonas* sp. GW456-R21	GCF_002901545.1	81.3	0.08	97.99
	*Pseudomonas* sp. efr-133-R2A-59	GCF_030209265.1	81.2	0.09	97.97
*P. aestiva* DGS32	*Pseudomonas oryzihabitans* RIT-PI-U	GCF_025642895.1	95.8	0.08	99.43
	*Pseudomonas oryzihabitans* UMB4614-CU331R	GCF_030228695.1	82.5	0.27	97.97
	*Pseudomonas* sp. SORGH_AS_0199	GCF_031453735.1	82.1	0	97.95
	*Pseudomonas psychrotolerans* KNF2016	GCF_014522265.1	81.3	0.07	97.90
	*Pseudomonas* sp. CBMAI 2609	GCF_029872515.1	81.6	0.4	97.87
	*Pseudomonas* sp. LA5	GCF_028640825.1	81.7	0.02	97.86
	*Pseudomonas* sp. BAV 4579	GCF_009765395.1	72.2	0.48	96.82
	*Pseudomonas oryzihabitans* MS8	GCF_003293465.1	71.8	0.43	96.80
	*Pseudomonas* sp. PS02302	GCF_029959585.1	72.4	0.43	96.80
	*Pseudomonas* sp. BAV 2493	GCF_009765535.1	72.2	0.47	96.76

Genomes are considered to be different strains of the same species only if they achieve ≥ 70% dDDH and ≥ 95% ANI and differ by less than 1% in GC% content. No strains were identified for *P. grandcourensis* or *P. helvetica*.

### Functional genome annotation reveals host-interaction and plant-beneficial features

**FIGURE 3:**
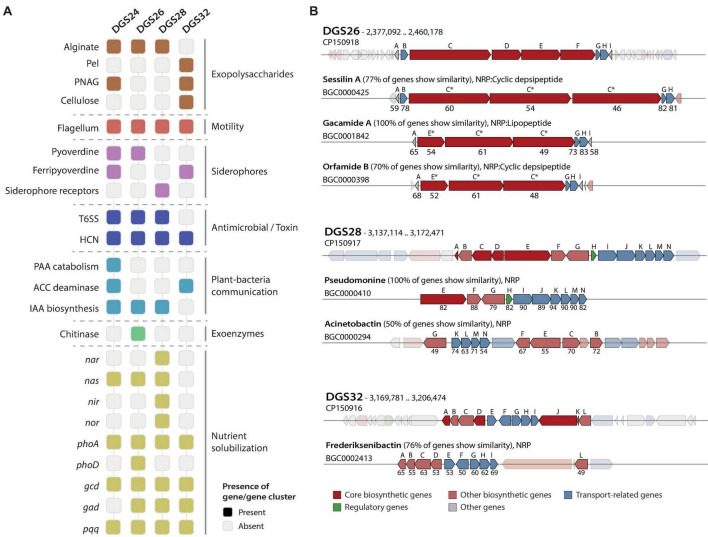
Host interaction and plant-beneficial characters identified by functional genome annotation. **(A)** Distribution of characters of interest in the four *Pseudomonas* genomes. Alginate biosynthesis: *alg8ADEFGJKLX*, pellicle formation (Pel): *pelABCDEFG*, poly-N-acetylglucosamine (PNAG): *pgaABCD*, cellulose biosynthesis: *bscAB+yhjQ*, flagellum: *flgFGHIJKL*+*fliC*+*flaG*+*fliDST*+*fleQSR*+*fliEFGHIJKLMN*+*flgEDCBAMN*+*motAB*, pyoverdine biosynthesis: *pvdAEHJMOPQSTY*, ferripyoverdine: *fpvA*, siderophore receptors: *pvcAB*, T6SS: *tssABCEFGHJKLM+clpV* and copies of *hcp* and *vrgG* genes, hydrogen cyanide (HCN) biosynthesis: *hcnABC*, phenylacetic acid (PAA) catabolism: *paaABCDEFGHIJK*, ACC deaminase: *acdS*, indole-3-acetic acid (IAA) biosynthesis: *iaaHM*, chitinase: *chiC*, nitrate reductase (*nar*): *narGHIJKLUX*, periplasmic nitrate reductase (*nas*): *nasATSED*, nitrite reductase (*nir*): *nirCDEFGHJLMNQS*, nitric oxide reductase (*nor*): *norBCD*, alkaline phosphatases: *phoA* and *phoD*, glucose dehydrogenase: *gcd*, gluconate dehydrogenase: *gad*, pyrroloquinoline quinone biosynthesis: *pqqABCDEF*. Gray squares represent the absence of the gene/gene cluster in the genome. **(B)** Comparison of secondary metabolite biosynthetic gene clusters encoding putative non-ribosomal peptides (NRP) with the most similar hits from the MIBiG database. Genes shown in low-opacity colors represent those with no sequence homology. Capital letters above genes indicate the order in which they appear in the genome of the strains characterized in this study. Letters above MIBiG reference gene clusters represent the gene to which they have the highest similarity, with percentages of BLAST sequence identity below. Asterisks indicate that multiple genes share homology, and only the percentages of BLAST sequence identity results with the highest bit scores are shown. Genes are colored according to their putative function.

Effective colonization of the rhizosphere environment by bacteria relies on key characteristics. Notably, motility and biofilm formation allow soil-dwelling bacteria to reach the roots, where they attach and colonize by developing microcolonies (Turnbull et al., 2001; Naseem et al., 2018; Blanco-Romero et al., 2024). The genomes of the four novel species characterized in this study were functionally annotated (Figure 3A, Supplementary Table 6), revealing that they harbor a complete set of genes encoding a single flagellar apparatus controlled by the *flhDC* master regulator operon (Soutourina and Bertin, 2003). This is consistent with the observation of flagellar structures by transmission electron microscopy (Figure 1). In addition, genes involved in the biosynthesis of biofilm components in *Pseudomonas* (Blanco-Romero et al., 2020) were found in the four genomes. These gene clusters included those required for the biosynthesis of the exopolysaccharides alginate (*algAD8KEXGJLF*), present in DGS24, DGS26 and DGS28, poly-N-acetyl-glucosamine (PNAG, *pgaABCD*), present in DGS26 and DGS28, and Pel (pellicle) (*pelABCDEFG*), present in DGS32 (Figure 3A, Supplementary Table 6). In addition, DGS32 harbors genes putatively involved in biosynthesis of cellulose (*bcsAB+yhjQ*), which has been suggested to play a role similar to PNAG in the structure of extracellular matrix components and biofilm formation (Lind et al., 2017; Blanco-Romero et al., 2020; Pentz and Lind, 2021).

Bacteria associated with plant roots contribute significantly to nutrient cycling, increasing the availability of plant-limiting nutrients, particularly phosphate and nitrogen (Hayat et al., 2010; Trivedi et al., 2020). The solubilization of inorganic phosphate by pseudomonads can be achieved by acidification of the environment, for which the enzymes glucose dehydrogenase and gluconate dehydrogenase, and the redox cofactor pyrroloquinoline quinone have previously been implicated (de Werra et al., 2009; Hayat et al., 2010). Genes predicted to encode these two enzymes (*gcd* and *gad*, respectively) and the biosynthetic gene cluster of pyrroloquinoline quinone (*pqqABCDEF*) are found in the DGS26, DGS28 and DGS32 genomes (Figure 3A, Supplementary Table 6). Furthermore, the genes for alkaline phosphatase PhoA, present in the four *Pseudomonas* genomes, and the additional alkaline phosphatase PhoD, found only in the DGS26 genome, suggest that these strains may contribute to improve phosphorus bioavailability to the plant. On the other hand, the DGS28 genome encodes two putative multimeric nitrate reductases (*nasASTED* and *narGHIJKLUX*) involved in the reduction of nitrate to nitrite (Richardson et al., 2001), a nitrite reductase (*nirCDEFGHIJLMNQS*) involved in the reduction of nitrite to nitric oxide (Kawasaki et al., 1997; Zumft, 1997), and a nitric oxide reductase (*norBCD*) involved in the reduction of nitric oxide to nitrous oxide (Zumft, 1997). The genomes of DGS24 and DGS26 contain only the *nas* gene cluster, while DGS32 does not contain any of these genes. The putative ability of these strains to solubilize phosphate and convert nitrogen species could have a positive impact on plant nutrition and growth.

Root-associated bacteria can produce compounds that influence the plant’s hormonal balance resulting in beneficial outcomes (Glick, 2005; Glick et al., 2007; Eichmann et al., 2021). Among these, the bacterial enzyme ACC deaminase attenuates plant responses to abiotic stresses by lowering the levels of the phytohormone ethylene (Glick, 2005), thereby conferring protection against moderate drought and salinity (Glick et al., 2007). We found the ACC deaminase gene (*acdS*) in the genomes of DGS24 and DGS32 (Figure 3A, Supplementary Table 6). In addition, bacteria can biosynthesize indole-3-acetic acid (IAA), the most prevalent auxin phytohormone in plants, which can exogenously stimulate root system development and favor plant nutrient uptake (Spaepen et al., 2007; Ali et al., 2009). The genes *iaaM* and *iaaH* encode the enzymes tryptophan 2-monooxygenase and indoleacetamide hydrolase that are responsible for the conversion of L-tryptophan to indole-3-acetamide (IAM) and then to IAA, respectively (Costacurta and Vanderleyden, 1995; Patten and Glick, 1996). Both genes were identified in strains DGS24, DGS26 and DGS28 (Figure 3A), making them candidates for promoting plant growth by stimulating the development of the root system (Spaepen et al., 2007; Ali et al., 2009). Phenylacetic acid (PAA) is another plant auxin involved in root elongation, enhancing lateral root formation and promoting overall plant growth (Cook, 2019), in addition to having antimicrobial activities against bacteria and yeast (Kim et al., 2004). The entire PAA catabolic gene cluster [*paaABCDEFGHIJKXYZ*, (Teufel et al., 2010)] was identified in the DGS24 genome, adding to its potential as a plant-beneficial inoculant by modulating the plant hormonal balance.

The synthesis of toxins by bacteria enables them to compete effectively with plant pathogens, thereby improving plant health by suppressing harmful organisms. Hydrogen cyanide is one of the most prevalent toxins in plant-beneficial *Pseudomonas* (Laville et al., 1998; Frapolli et al., 2012; Garrido-Sanz et al., 2021), which exhibits strong antimicrobial properties against a variety of plant pathogens, including fungi such as *Thielaviopsis basicola* (Voisard et al., 1989; Ramette et al., 2006). Genes for the biosynthesis of hydrogen cyanide (*hcnABC*) were found in the genome of all four strains studied (Figure 3A). In addition, bacteria can also inject specific toxic effectors into target prokaryotic or eukaryotic cells using the type VI secretion system (T6SS), which is commonly found in biocontrol bacteria (Bernal et al., 2017; Durán et al., 2021). The genomes of DGS24, DGS26 and DGS28 encode putative structural components of the T6SS (*tssABCEFGHJKLM+ClpV*, Supplementary Table 6). Two complete gene clusters were found in DGS24 and DGS28, putatively encoding two different T6SSs. Multiple putative copies of the *hcp* gene, encoding the Hcp T6SS structural tube (Bernal et al., 2018) were found in the three genomes: two copies in strain DGS24, four copies in DGS26, and seven copies in DGS26 (Supplementary Table 6). Moreover, putative *vgrG* genes encoding the piercing device that caps the Hcp tube were found in the three genomes: two copies in DGS24, six copies in DGS26, and seven copies in DGS28. The presence of T6SSs in three of the genomes suggests that they could efficiently target prokaryotic or eukaryotic cells, allowing them to compete with other rhizosphere bacteria or pathogens.

Secondary metabolite biosynthetic gene clusters (BGCs) were predicted in the genomes of the four strains, and included non-ribosomal peptides (NRPs), polyketides (PKs) and ribosomally synthesized and post-transcriptionally modified peptides (RiPPs, Supplementary Table 7). Although most of the predicted BGCs had low homology to known metabolites, DGS26, DGS28 and DGS32 contained NRPs with > 75% similarity to known clusters (Figure 3B, Supplementary Table 7). An 84.3 kbp region in the DGS26 genome harbors four genes similar to the BGCs of sessilin A, gacamide A, and orfamide B, which are potent cyclic lipopeptides/depsipeptides involved in fungal antagonism (D’aes et al., 2014; Jahanshah et al., 2019; Oni et al., 2019). Similarly, a 49.1 kbp region in the DGS28 genome harbors genes identical to those reported for the biosynthesis of the NRP pseudomonine in *Pseudomonas* sp. WCS374, a siderophore involved in iron acquisition (Mercado-Blanco et al., 2001). Finally, a 54 kbp region in the DGS32 genome carries genes very similar to those for the biosynthesis of frederiksenibactin, another siderophore involved in iron acquisition (Stow et al., 2021). NRP-related siderophores in *Pseudomonas* are known to have antimicrobial activity against fungi and oomycetes (Buysens et al., 1996; Kaplan et al., 2021; Grosse et al., 2023).

The features identified in the genomes of the four novel species described in this study highlight the putative potential of these bacteria to colonize the rhizosphere environment, promote plant growth, and potentially antagonize plant pathogens.

### Phenotypic characterization supports the species identity of the four novel *Pseudomonas*

**FIGURE 4:**
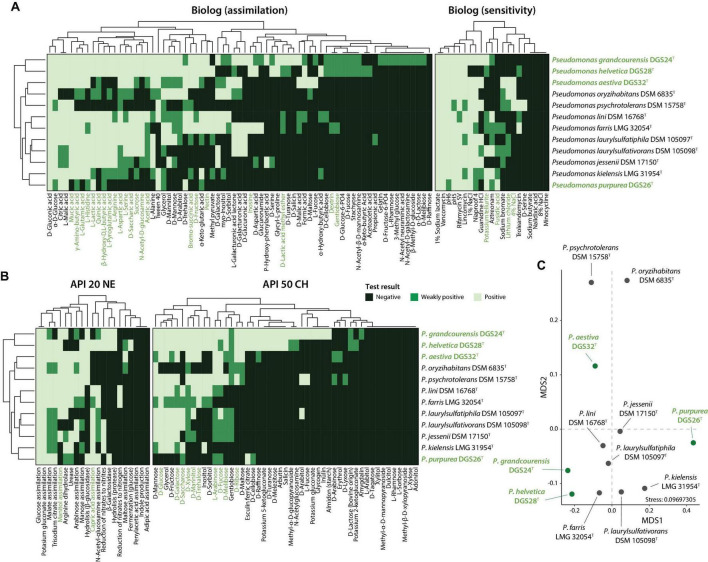
Phenotypic characterization of the four *Pseudomonas* described in this study (highlighted in green) and their closest relatives. **(A)** Biolog or **(B)** API strip results were clustered according to complete linkage of Euclidean distances and are presented as heatmaps. **(C)** Non-metric multidimensional scaling (NMDS) ordination analysis of the distinctive phenotypic traits. The phenotypic tests driving the observed distribution patterns in the ordination plot are indicated in green in panels **(A)** and **(B)**, with a *P* value ≤ 0.05 (*envfit*, 999 permutations).

Phenotypic differences between the four novel species described here and their closest type strains were evaluated by comparing the results of the API 50 CH, API 20 NE and Biolog GEN III tests. The results are summarized in Figures 4A, B and Table 4, and the complete list of results for all tests can be found in Supplementary Table 4. Of the 161 tests performed (92 Biolog, 20 API 20 NE and 49 API 50 CH), only a few showed relevant differences. This was expected as the strains are closely related and belong to the genus *Pseudomonas*.

**TABLE 4:** Phenotypic characterization of the four *Pseudomonas* strains described in this study and their closest relatives. Strains: 1 = *P. grandcourensis* DGS24^T^, 2 = *P. jessenii* DSM 17150^T^, 3 = *P. laurylsulfatiphila* DSM 105097^T^, 4 = *P. laurylsulfativorans* DSM 105098^T^, 5 = *P. purpurea* DGS26^T^, 6 = *P. kielensis* LMG 31954^T^, 7 = *P. lini* DSM 16768^T^, 8 = *P. helvetica* DGS28^T^, 9 = *P. farris* LMG 32054^T^, 10 = *P. aestiva* DGS32^T^, 11 = *P. psychrotolerans* DSM 15758^T^, 12 = *P. oryzihabitans* DSM 6835^T^, 13 = *P. benzopyrenica* BaP3^T^.

	Strains												
Characteristic	1	2	3	4	5	6	7	8	9	10	11	12	13
**Oxidation of (API 50 CH)**													
L-Arabinose	+	w	w	w	w	-	w	+	-	+	+	+	+
D-Ribose	+	−	−	−	−	−	−	+	−	+	+	w	+
D-Xylose	+	w	w	w	−	−	w	+	w	+	+	w	+
D-Glucose	+	+	+	+	−	+	+	+	+	+	+	−	+
Inositol	+	−	−	−	−	+	+	+	w	−	+	−	+
D-Mannitol	+	w	+	−	−	−	+	+	w	+	+	−	+
D-Sorbitol	+	−	−	−	−	−	+	+	w	−	+	−	+
Arbutin	+	−	−	−	−	−	−	+	−	−	−	−	nd
Esculin ferric citrate	+	−	−	−	+	−	−	+	−	−	−	−	nd
Salicin	+	−	−	−	−	−	−	+	−	−	−	−	nd
D-Cellobiose	+	−	−	−	w	−	−	+	−	−	−	−	nd
D-Maltose	+	−	−	−	−	−	−	+	−	+	w	w	nd
D-Melibiose	+	w	w	w	−	−	−	+	−	w	+	−	nd
D-Saccharose (sucrose)	+	−	+	+	w	+	+	+	w	−	−	−	−
D-Melezitose	+	−	−	−	−	−	−	+	−	−	−	−	nd
D-Raffinose	+	−	−	−	−	−	−	+	−	w	−	−	nd
D-Turanose	+	−	−	−	−	−	−	+	−	−	−	−	nd
Potassium 5-ketogluconate	+	−	−	−	−	−	−	+	−	−	−	−	nd
**Assimilation of (Biolog)**													
D-Salicin	+	−	−	−	−	-	−	−	−	−	−	−	nd
N-Acetyl-D-glucosamine	+	w	+	+	−	−	+	+	+	−	−	−	nd
D−Mannose	+	w	+	−	−	−	w	+	w	w	−	+	nd
D-Fructose	+	−	−	−	−	−	w	+	+	+	−	−	nd
D-Galactose	+	+	−	+	−	−	+	−	+	w	−	+	nd
Inosine	+	−	−	−	−	−	−	+	+	−	−	−	+
*myo*-Inositol	w	−	−	−	−	w	+	−	+	w	−	+	nd
D-Aspartic acid	w	w	−	−	−	−	−	+	+	−	−	−	nd
D-Serine	w	w	−	−	−	−	−	+	−	−	−	−	+
Glycyl-L-proline	+	−	−	−	−	−	−	+	−	−	−	−	nd
L-Glutamic acid	+	+	+	+	−	+	+	+	+	+	−	w	nd
L-Histidine	+	+	+	+	w	+	+	+	w	+	−	−	+
L-Serine	+	w	+	+	−	w	w	+	+	+	−	w	+
Pectin	+	−	−	−	−	−	+	+	+	−	−	−	nd
D-Galacturonic acid	+	−	+	+	−	−	−	−	+	+	−	w	+
L-Galacturonic acid lactone	+	−	+	+	−	−	−	w	+	+	−	w	+
D-Lactic acid methyl ester	+	−	−	−	−	−	w	w	w	−	−	−	nd
α-Keto-glutaric acid	+	−	w	−	w	+	+	+	+	w	−	−	+
D-Malic acid	+	−	−	−	−	−	w	−	w	+	−	−	+
Bromo-succinic acid	+	−	w	−	−	w	+	+	+	+	w	−	+
Tween 40	+	−	−	−	+	+	+	+	−	+	+	w	+
Acetic acid	+	w	w	w	−	−	+	+	w	w	−	−	+
**Sensitivity to (Biology)**													
Fusidic acid	−	−	−	w	+	w	−	−	w	−	−	−	nd
Guanidine HCl	+	+	+	w	−	w	−	−	−	+	+	−	nd
Niaproof 4	+	+	+	+	w	w	+	−	w	+	+	+	nd
Potassium tellurite	+	−	−	+	+	+	w	w	w	−	−	−	nd
Aztreonam	w	−	w	+	w	+	+	−	w	−	−	−	nd
**API 20 NE enzymatic activities**													
Reduction of nitrates to nitrites	+	+	−	+	−	+	−	+	−	−	−	−	nd
Fermentation (glucose)	−	−	−	−	−	−	−	+	−	−	−	−	nd
Arginine dihydrolase	+	w	+	−	w	+	+	−	+	+	+	+	nd
Urease	−	+	-	−	w	+	+	−	+	+	+	+	nd
Hydrolysis (β-glucosidase)	+	−	−	−	+	−	−	+	−	+	+	+	nd
Hydrolysis (protease)	+	−	−	−	−	−	−	+	−	−	−	−	nd
β-Galactosidase	+	−	−	−	+	-	−	+	−	−	−	−	nd

Only the most relevant results are shown. The complete results can be found in Supplementary Table 4. For assimilation and oxidation of carbon compounds: positive (+), negative (−), weakly positive (w). For sensitivity: insensitive (+, can grow in its presence) sensitive (−, cannot grow in its presence). For *P. benzopyrenica* BaP3^T^, results are based on Dong et al. (2023), not defined (nd).

*P. grandcourensis* DGS24^T^ was compared with the closely related type strains *P. jessenii* DSM 17150^T^, *P. laurylsulfatiphila* DSM 105097^T^, and *P. laurylsulfativorans* DSM 105098^T^ and showed a differential oxidation (API 50 CH) of 21 compounds: D-ribose, inositol, D-sorbitol, methyl-α-D-glucopyranoside, N-acetylglucosamine, arbutin, esculin ferric citrate, salicin, D-cellobiose, D-maltose, D-trehalose, inulin, D-melezitose, D-raffinose, starch, glycogen, D-turanose, L-fucose, potassium gluconate and potassium 5-ketogluconate. The strain also exhibited β-glucosidase and β-galactosidase activities (API 20 NE). Conversely, *P. grandcourensis* DGS24^T^ was the only of the three closely related strains that was unable to assimilate trisodium citrate and capric acid (API 20 NE).

*P. purpurea* DGS26^T^ was compared with the two closest related type strains, *P. kielensis* LMG 31954^T^ and *P. lini* DSM 16768^T^. The newly identified species *P. purpurea* exhibited differentially positive β-galactosidase, β-glucosidase and esculin ferric citrate oxidation activities (API 20 NE and API 50 CH). Conversely, *P. kielensis* LMG 31954^T^ and *P. lini* DSM 16768^T^ were able to oxidize (API 50 CH) glycerol, D-galactose, D-glucose, D-fructose, D-mannose, inositol and D-saccharose and to grow (Biolog) in the presence of sucrose, α-D-glucose, *myo*-inositol, glycerol, L-alanine, L-arginine, L-aspartic acid, L-glutamic acid, L-histidine, L-pyroglutamic acid, L-serine, mucic acid, methyl pyruvate, bromo-succinic acid, and α-hydroxy-butyric acid while *P. purpurea* DGS26^T^ was not. *P. lini* DSM 16768^T^ was the only strain able to utilize (Biolog) D-cellobiose, N-acetyl-D-glucosamine, D-mannose, D-fructose, D-galactose, D-sorbitol, D-mannitol, D-arabitol, pectin, quinic acid, D-saccharic acid, D-lactic acid methyl ester, D-malic acid, acetic acid, formic acid and reduce nitrates to nitrogen (API 20 NE).

*P. helvetica* DGS28^T^ was compared with the two closest type strains, *P. farris* LMG 32054^T^ and *P. kielensis* LMG 31954^T^. *P. helvetica* DGS28^T^ was able to differentially oxidize (API 50 CH) L-arabinose, D-ribose, methyl-α-D-glucopyranoside, N-acetylglucosamine, arbutin, esculin ferric citrate, salicin, D-cellobiose, D-maltose, D-lactose, D-melibiose, D-melezitose, D-raffinose, gentiobiose, D-turanose, D-fucose, potassium 2-ketogluconate and potassium 5-ketogluconate. In addition, DGS28 assimilated (Biolog) D-cellobiose, D-turanose, stachyose, N-acetyl- β -D-mannosamine, D-fucose, D-glucose-6-PO_4_, D-serine, glycyl-L-proline, α-keto-butyric acid, acetoacetic acid and formic acid, and showed β-galactosidase and β-glucosidase enzymatic activities (API 20 NE), while *P. farris* LMG 32054^T^ and *P. kielensis* LMG 31954^T^ showed negative results in all the mentioned tests. On the contrary, *P. helvetica* DGS28^T^ tested negative for the assimilation of *myo*-inositol (Biolog), while the two closely related strains could assimilate this compound. *P. helvetica* DGS28^T^ also failed to grow on 1% NaCl, fusidic acid, niaproof 4 and aztreonam while *P. farris* LMG 32054^T^ and *P. kielensis* LMG 31954^T^ did.

*P. aestiva* DGS32^T^ was compared with the three closest type strains, *P. psychrotolerans* DSM 15758^T^, *P. oryzihabitans* DSM 6835^T^ and *P. benzopyrenica* BaP3^T^, which was recently described (Dong et al., 2023). Among the phenotypic characteristics, *P. aestiva* DGS32^T^ was able to differentially oxidize (API 50 CH) erythrol and D-raffinose and assimilate (Biolog) sucrose, D-fructose and L-fucose. *P. aestiva* DGS32^T^, *P. psychrotolerans* DSM 15758^T^ and *P. benzopyrenica* BaP3^T^ showed a positive ability to oxidize (API 50 CH) glycerol, D-arabinose, D-galactose, D-glucose, D-fructose, D-mannitol, D-trehalose, assimilate (Biolog) mucic acid, citric acid, L-malic acid and bromo-succinic acid, while *P. oryzihabitans* DSM 6835^T^ could not. Both *P. aestiva* DGS32^T^ and *P. benzopyrenica* BaP3^T^ were the only ones able to assimilate L-histidine, α-keto-glutaric acid, D-malic acid, acetic acid and formic acid.

The differences between the type strains were further investigated by an NMDS ordination analysis, which shows a clear distinction between the type strains of the twelve species studied (Figure 4C). The differences were mainly supported by the differential oxidation of certain sugars, including glucose, galactose, saccharose or xylose, and the use of amino acids such as glutamic acid, histidine, arginine, serine, glutamic acid, and aspartic acid.

### Promotion of wheat growth and antagonism to phytopathogens

**FIGURE 5:**
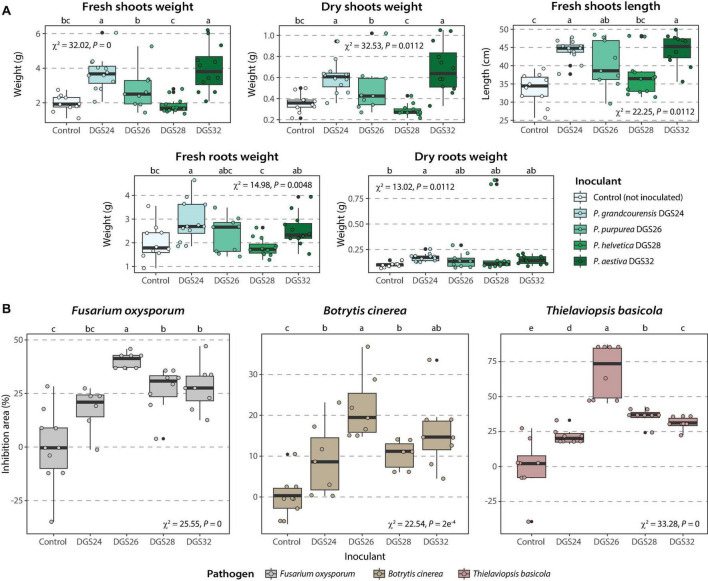
Promotion of wheat growth induced by inoculation of the four strains analyzed in this study and phytopathogen inhibition. **(A)** Promotion of wheat growth. Box plots show the different parameters measured for each strain. Strains were inoculated onto 2-day-old wheat seedlings at an initial concentration of ∼3⋅10^6^ CFU/mL per plant. **(B)** Inhibition of three phytopathogens in dual culture assays. Boxplots show the different percentage of inhibition of mycelial growth measured per strain. The percent inhibition area (*Ai*) was calculated using the formula: *Ai* (%) = (*Ac*–*At*) / *Ac* 100, where *Ac* is the mean area of the pathogen in control plates and *At* is the area of the pathogen in test plates (inoculated with bacteria). Differences between groups were assessed using the Kruskal-Wallis rank sum test, with Fisher’s least significant difference post hoc test. *P* values were corrected using the false discovery rate. Different letters indicate significant differences between groups at a *P* value ≤ 0.05.

The ability of the four strains to promote wheat growth was evaluated by inoculating cells onto 2-day-old wheat seedlings. Plant biomass after 21 days was increased in the presence of strains DGS24 and DGS32 (Figure 5A, Supplementary Table 5). Specifically, the inoculation with each strain doubled the fresh shoot biomass and resulted in an increase in plant length of ∼28.5%. The increase in root biomass was less pronounced, but also observed. Inoculation with strains DGS26 and DGS28 resulted in more discrete differences. Mean fresh shoot and root weights were increased by DGS26 inoculation, as was plant length. DGS28 only increased the mean values of shoot length and root dry weight. The pronounced direct growth promotion observed following inoculation with DGS24 and DGS32 could be related to these strains’ putative ability to modulate plant hormones and phosphate solubilization (Figure 3A).

In addition, the ability of the four strains to inhibit the growth of three fungal plant pathogens, *Fusarium oxysporum*, *Botrytis cinerea*, and *Thielaviopsis basicola*, was evaluated using dual culture assays. Strains DGS26, DGS28 and DGS32 significantly inhibited the three pathogens, with DGS26 showing the highest antagonistic ability with up to 50% inhibition of *F. oxysporum*, 75% for *T. basicola* and about 20% for *B. cinerea* (Figure 5B). DGS28 and DGS32 exhibited similar pathogen growth inhibition, around 30% for *F. oxysporum* and *T. basicola*, and 15% for *B. cinerea.* Finally, DGS24 did not significantly inhibit *F. oxysporum*, and had a discrete effect on *B. cinerea* and *T. basicola*, with 10% and 25% inhibition, respectively. The ability of DGS26 to significantly antagonize the growth of the three fungal pathogens could be attributed to the predicted biosynthesis of cyclic lipopeptides with similarity to sessilin A, gacamicin A and orfamide B (Figure 3B), which are known for their potent antimicrobial and biosurfactant activities (D’aes et al., 2014; Jahanshah et al., 2019; Oni et al., 2019). In the case of DGS28 and DGS32, the two putative NRP siderophores encoded in their genomes, which show high similarity to pseudomonine (Mercado-Blanco et al., 2001) in DGS28 and frederiksenibactin (Stow et al., 2021) in DGS32 (Figure 3B), could be involved in their fungal antagonism, as other NRP siderophores produced by *Pseudomonas* have antifungal activity (Buysens et al., 1996; Ho et al., 2021; Kaplan et al., 2021; Grosse et al., 2023). The presence of the hydrogen cyanide biosynthetic cluster in all strains may also contribute to the observed antagonistic phenotype of the strains (Voisard et al., 1989; Laville et al., 1998).

These results highlight the plant-beneficial potential of the four novel species on wheat. Direct inoculation with DGS24 and DGS32 strains significantly increased plant biomass, while DGS26, DGS28 and DGS32 demonstrated effective antagonism against three fungal phytopathogens.

### Description of *Pseudomonas grandcourensis* sp. nov.

*Pseudomonas grandcourensis* (grand.cou.ren.sis. N.L. neut. adj. *grandcourensis*, from Grandcour, Switzerland, referring to the region where soil was used to grow wheat plants and isolate the type strain DGS24^T^).

Cells of this species are aerobic, Gram-negative, non-spore-forming, motile by means of a single polar flagellum, and rod-shaped, 1.5 μm wide and 2.9 μm long. On R2A medium, cells can grow between 20 and 35°C (optimum 28°C), and colonies are white and mucoid, with diffuse edges after 24 h of growth. They produce a pale-yellow pigment. Results obtained with Biolog Gen III microplates indicate that the cells can use the following substrates as carbon and energy sources: sucrose, D-turanose, D-salicin, N-acetyl-D-glucosamine, α-D-glucose, D-mannose, D-fructose, D-galactose, inosine, D-mannitol, D-arabitol, glycerol, glycyl-L-proline, L-alanine, L-arginine, L-aspartic acid, L-glutamic acid, L-histidine, L-pyroglutamic acid, L-serine, pectin, D-galacturonic acid, L-galacturonic acid lactone, D-gluconic acid, D-glucuronic acid, mucic acid, quinic acid, D-saccharic acid, *p*-hydroxy-phenylacetic acid, D-lactic acid methyl ester, L-lactic acid, citric acid, α-keto-glutaric acid, D-malic acid, L-malic acid, bromo-succinic acid, Tween 40, γ-amino-butyric acid, β-hydroxy-D, L-butyric acid and acetic acid. All other substrates in the Biolog GEN III panel did not or only weakly support cell growth. Based on Biolog Gen III sensitivity assays, cells of this species can grow between pH 5 and pH 7, 1% NaCl, and with 1% sodium lactate. Cells are resistant to rifamycin SV, lincomycin, guanidine HCl, niaproof 4, vancomycin and potassium tellurite. Cell growth was inhibited by all other compounds tested in the Biolog GEN III sensitivity assays. Results obtained with API 50 CH and API 20 NE strips indicate that cells of this species oxidize the following substrates: glycerol, L-arabinose, D-ribose, D-xylose, D-galactose, D-glucose, D-fructose, D-mannose, inositol, D-mannitol, D-sorbitol, methyl-α-D-glucopyranoside, N-acetylglucosamine, arbutin, esculin ferric citrate, salicin, D-cellobiose, D-maltose, D-melibiose, D-saccharose, D-trehalose, inulin, D-melezitose, D-raffinose, starch, glycogen, D-turanose, D-fucose, L-fucose, D-arabitol, potassium gluconate and potassium 5-ketogluconate. Cells of this species exhibited the following enzymatic activities detected by API 20 NE: reduction of nitrates to nitrites, arginine dihydrolase, hydrolysis (β-glucosidase and protease) and β-galactosidase. The genome of *P. grandcourensis* DGS24^T^ consists of a circular chromosome of 6.76 Mbp, 59.7% G+C, 5,967 protein-coding genes, seven copies of the 16S rRNA and 23S rRNA genes, eight copies of the 5S rRNA gene, and 73 tRNAs for the transfer of 39 different amino acids. The whole genome sequence of *P. grandcourensis* DGS24^T^ has been deposited at NCBI and is publicly available at NCBI GeneBank accs. no. CP150919.

The type strain is DGS24^T^ ( = DSM 117501^T^ = CECT 31011^T^), isolated in 2021 from the rhizosphere of wheat.

### Description of *Pseudomonas purpurea* sp. nov.

*Pseudomonas purpurea* (pur.pu’re.a. L. fem. adj. *purpurea*, purple-color, referring to the color of the diffusible pigment produced by the type strain DGS26^T^).

Cells of this species are aerobic, Gram-negative, non-spore-forming, motile by means of two polar flagella, and rod-shaped, 1.6 μm wide and 3.3 μm long. In R2A medium, cells can grow between 20 and 28°C (optimum 28°C), and colonies are translucent and compact. After 48 h of growth, the cells produce a diffusible light purple pigment. Results obtained with Biolog Gen III microplates indicate that cells can use the following substrates as carbon and energy sources: D-trehalose, α-D-glucose, glycerol, L-histidine, D-gluconic acid, L-lactic acid, citric acid, α-keto-glutaric acid, L-malic acid, Tween 40, γ-amino-butyric acid and β-hydroxy-D, L-butyric acid. All other substrates in the Biolog GEN III panel did not support cell growth. Based on Biolog Gen III sensitivity assays, cells of this species can grow between pH 5 and pH 7, with 1% NaCl, and with 1% sodium lactate, and are resistant to fusidic acid, rifamycin SV, lincomycin, niaproof 4, vancomycin, potassium tellurite and aztreonam. Cell growth was inhibited by all other compounds tested in the Biolog GEN III sensitivity assays. Results obtained with API 50 CH and API 20 NE strips indicate that cells of this species oxidize L-arabinose, esculin ferric citrate, D-cellobiose, D-saccharose and D-fucose. Cells of this species exhibited the following enzymatic activities as detected by API 20 NE: arginine dihydrolase, urease, hydrolysis (β-glucosidase) and β-galactosidase. In API 20 NE cells grow on the following substrates: glucose, N-acetyl-glucosamine, potassium gluconate, capric acid, malate and trisodium citrate. The genome of *P. purpurea* DGS26^T^ consists of a circular chromosome of 5.93 Mbp, 59.9% G+C, 5,388 protein-coding genes, seven copies of the 16S rRNA and 23S rRNA genes, eight copies of the 5S rRNA gene, and 75 tRNAs for the transfer of 38 different amino acids. The whole genome sequence of *P. purpurea* DGS26^T^ has been deposited at NCBI and is publicly available at NCBI GeneBank accs. no. CP150918.

The type strain is DGS26^T^ ( = DSM 117502^T^ = CECT 31012^T^), isolated in 2021 from the rhizosphere of wheat.

### Description of *Pseudomonas helvetica* sp. nov.

*Pseudomonas helvetica* (hel.ve’ti.ca. N.L. fem. adj. *helvetica*, of or belonging to the Helvetians or to Helvetia, the neo-Latin name of Switzerland where the type strain DGS28^T^ was isolated).

Cells of this species are aerobic, Gram-negative, non-spore-forming, motile by means of two polar flagella, and rod-shaped, 1.6 μm wide and 3.1 μm long. In R2A medium, cells can grow between 20 and 35°C (optimum 28°C), and colonies are small, compact, and pale-yellow. Old colonies show a green pigment in the center of the colony. Results obtained with Biolog Gen III microplates indicate that the cells can use the following substrates as carbon and energy sources: D-trehalose, sucrose, N-acetyl-D-glucosamine, α-D-glucose, D-mannose, D-fructose, inosine, D-mannitol, D-arabitol, glycerol, D-aspartic acid, D-serine, glycyl-L-proline, L-alanine, L-arginine, L-aspartic acid, L-glutamic acid, L-histidine, L-pyroglutamic acid, L-serine, pectin, D-gluconic acid, mucic acid, quinic acid, D-saccharic acid, methyl pyruvate, L-lactic acid, citric acid, α-keto-glutaric acid, L-malic acid, bromo-succinic acid, Tween 40, γ-amino-butyric acid, α-hydroxy-butyric acid, β-hydroxy-D,L-butyric acid, acetic acid, and formic acid. All other substrates in the Biolog GEN III panel did not or only weakly support cell growth. Based on Biolog Gen III sensitivity assays, cells of this species can grow between pH 5 and pH 7, with 1% sodium lactate, and are resistant to rifamycin SV, lincomycin and vancomycin. Cell growth was inhibited by all other compounds tested within the Biolog GEN III sensitivity assays. Results obtained with API 50 CH and API 20 NE strips indicate that cells of this species can oxidize the following substrates: glycerol, L-arabinose, D-ribose, D-xylose, D-galactose, D-glucose, D-fructose, D-mannose, inositol, D-mannitol, D-sorbitol, arbutin, esculin ferric citrate, salicin, D-cellobiose, D-maltose, D-melibiose, D-saccharose, D-trehalose, D-melezitose, D-raffinose, D-turanose, potassium 5-ketogluconate, and assimilate potassium gluconate, capric acid, malate and trisodium citrate. Cells of this species exhibited the following enzymatic activities as detected by API 20 NE: reduction of nitrates to nitrites, fermentation of glucose and hydrolysis (β-glucosidase and protease). The genome of *P. helvetica* DGS28^T^ consists of a circular chromosome of 6.44 Mbp, 58.8% G+C, 5,857 protein-coding genes, six copies of the 16S rRNA and 23S rRNA genes, seven copies of the 5S rRNA gene, and 69 tRNAs for the transfer of 38 different amino acids. The whole genome sequence of *P. helvetica* DGS28^T^ has been deposited at NCBI and is publicly available at NCBI GeneBank accs. no. CP150917.

The type strain is DGS28^T^ ( = DSM 117503^T^ = CECT 31013^T^), isolated in 2021 from the rhizosphere of wheat.

### Description of *Pseudomonas aestiva* sp. nov.

*Pseudomonas aestiva* (aes.ti.va. L. fem. adj. *aestiva*, of summer, referring to the species name of the host plant (*Triticum aestivum*) from which the type strain DGS32^T^ was isolated).

Cells of this species are aerobic, Gram-negative, non-spore-forming, motile by means of a single polar flagellum, and rod-shaped, 1.6 μm wide and 3.4 μm long. In R2A medium, cells can grow between 20 and 28°C (optimum 28°C), and colonies are small, compact, and orange. Results obtained with Biolog Gen III microplates indicate that cells can use the following substrates as carbon and energy sources: D-maltose, D-trehalose, α-D-glucose, D-fructose, L-fucose, D-sorbitol, L-alanine, L-aspartic acid, L-glutamic acid, L-histidine, L-serine, D-galacturonic acid, L-galacturonic acid lactone, D-gluconic acid, mucic acid, D-saccharic acid, citric acid, D-malic acid, L-malic acid, bromo-succinic acid, Tween 40, γ-amino-butyric acid and formic acid. All other substrates in the Biolog GEN III panel did not or only weakly support cell growth. Based on Biolog Gen III sensitivity assays, cells of this species can grow between pH 5 and 7, between 1% and 4% NaCl, with 1% sodium lactate, and are resistant to rifamycin SV, lincomycin, guanidine HCl, niaproof 4, vancomycin, lithium chloride and sodium bromate. Cell growth was inhibited by all other compounds tested within the Biolog GEN III sensitivity assays. Results obtained with API 50 CH and API 20 NE strips indicate that cells of this species oxidize the following substrates: glycerol, L-arabinose, D-ribose, D-xylose, D-galactose, D-glucose, D-mannitol, D-maltose, D-trehalose, D-fucose, and assimilate mannose, potassium gluconate, malate, and trisodium citrate. Cells of this species exhibited the following enzymatic activities as detected by API 20 NE: arginine dihydolase, urease, hydrolysis (β-glucosidase). The genome of *P. aestiva* DGS32^T^ consists of a circular chromosome of 4.98 Mbp, 65.9% G+C, 4,500 protein-coding genes, five copies of the 16S rRNA, 23S rRNA and 5S rRNA genes, and 74 tRNAs for the transfer of 40 different amino acids. The whole genome sequence of *P. aestiva* DGS32^T^ has been deposited at NCBI and is publicly available at NCBI GeneBank accs. no. CP150916.

The type strain is DGS32^T^ ( = DSM 117504^T^ = CECT 31014^T^), isolated in 2021 from the rhizosphere of wheat.

### Emended description of *Pseudomonas alvandae* (Girard et al., 2021)

*Pseudomonas alvandae* (al.van’dae. N.L. gen. n. *alvandae*, from Alvand, a wheat cultivar).

The description is as reported by Girard et al. (2021).

The type strain is SWRI17^T^ ( = LMG 32056^T^ = CFBP 8869^T^). The name *Pseudomonas canavaninivorans* (Hauth et al., 2022) type strain HB002^T^ ( = DSM 112525^T^ = LMG 32336^T^) is a later heterotypic synonym.

### Emended description of *Pseudomonas oryzihabitans* (Kodama et al., 1985)

*Pseudomonas oryzihabitans* (o.ry.zi’ha.bi.tans. L. fem. n. *oryza* rice; L. fem. adj. *habitans* inhabiting; M. L. fem. adj. *oryzihabitans* rice inhabiting).

The description is as reported by Kodama et al. (1985).

The type strain is KS0036^T^ ( = L-l^T^ = AJ 2197^T^ = IAM 1568^T^ = JCM 2592^T^). The name *Pseudomonas psychrotolerans* (Hauser et al., 2004) type strain C36^T^ ( = LMG 21977^T^ = DSM 15758^T^) is a later heterotypic synonym.

### Emended description of *Pseudomonas ogarae* (Garrido-Sanz et al., 2021)

*Pseudomonas ogarae* (o.ga.rae. N.L. gen. n. *ogarae*, after Fergal O’Gara, Irish microbiologist who isolated the strain F113^T^ and defined its first plant growth-promoting features).

The description is as reported by Garrido-Sanz et al. (2021).

The type strain is F113^T^ ( = DSM 112162^T^ = CECT 30235^T^). The name *Pseudomonas zarinae* (Girard et al., 2021) type strain SWRI108^T^ ( = CFBP 8856^T^ = LMG 32043^T^) is a later heterotypic synonym.

## Conclusion

In this work, we have reported the characterization of four novel *Pseudomonas* species isolated from the rhizosphere of wheat, which expands the known diversity of crop-associated bacteria. Our results demonstrate that the four isolated strains belong to novel species within the genus *Pseudomonas* by exhibiting noticeable genotypic and phenotypic differences compared to their closest relatives. In addition, two of the strains represent a novel phylogenomic subgroup within the *Pseudomonas fluorescens* species complex. The genomes of the four novel species harbor host-interaction and plant-beneficial characteristics that make them interesting for use as inoculants in agriculture. These include characters important for efficient colonization of the rhizosphere environment, such as flagella and biofilm formation components. In addition, their ability to transform nitrogen species and putatively solubilize phosphate may contribute to improved plant nutrition. Similarly, their potential to modulate the plant hormone balance through ACC deaminase activity or plant auxin metabolism could contribute to increased crop productivity. Indeed, direct inoculation of DGS24 and DGS32 doubled the shoot biomass of wheat plants, while DGS26, DGS28 and DGS32 efficiently antagonized three fungal phytopathogens. These results highlight the potential of the four novel species for use as biofertilizers and biocontrol agents in agriculture and add to the known diversity of bacteria associated with the wheat rhizosphere.

## Data availability statement

The datasets presented in this study can be found in online repositories. The names of the repository/repositories and accession number(s) can be found in the article/Supplementary material.

## Author contributions

NP: Formal analysis, Investigation, Methodology, Visualization, Writing−original draft, Writing−review and editing. CK: Funding acquisition, Project administration, Supervision, Validation, Writing−review and editing. DG-S: Conceptualization, Data curation, Formal analysis, Funding acquisition, Investigation, Methodology, Project administration, Resources, Software, Supervision, Validation, Visualization, Writing−original draft, Writing−review and editing.

## Footnotes

1
https://lpsn.dsmz.de/genus/pseudomonas

## References

[B1] Ali B. Sabri A. N. Ljung K. Hasnain S. (2009). Auxin production by plant associated bacteria: Impact on endogenous IAA content and growth of *Triticum aestivum* L. *Lett. Appl. Microbiol.* 48 542–547. 10.1111/j.1472-765X.2009.02565.x 19220737

[B2] Bakker P. A. H. M. Pieterse C. M. J. de Jonge R. Berendsen R. L. (2018). The soil-borne legacy. *Cell* 172 1178–1180. 10.1016/j.cell.2018.02.024 29522740

[B3] Barahona E. Navazo A. Garrido-Sanz D. Muriel C. Martínez-Granero F. Redondo-Nieto M. (2016). *Pseudomonas fluorescens* F113 can produce a second flagellar apparatus, which is important for plant root colonization. *Front. Microbiol.* 7:1471. 10.3389/fmicb.2016.01471 27713729 PMC5031763

[B4] Bernal P. Allsopp L. P. Filloux A. Llamas M. A. (2017). The *Pseudomonas putida* T6SS is a plant warden against phytopathogens. *ISME J.* 11 972–987. 10.1038/ismej.2016.169 28045455 PMC5363822

[B5] Bernal P. Llamas M. A. Filloux A. (2018). Type VI secretion systems in plant-associated bacteria. *Environ. Microbiol.* 20 1–15. 10.1111/1462-2920.13956 29027348 PMC5813230

[B6] Blanco-Romero E. Garrido-Sanz D. Durán D. Rybtke M. Tolker-Nielsen T. Redondo-Nieto M. (2024). Role of extracellular matrix components in biofilm formation and adaptation of *Pseudomonas ogarae* F113 to the rhizosphere environment. *Front. Microbiol.* 15:1341728. 10.3389/fmicb.2024.1341728 38333580 PMC10850567

[B7] Blanco-Romero E. Garrido-Sanz D. Rivilla R. Redondo-Nieto M. 8r4Martín M. (2020). In silico characterization and phylogenetic distribution of extracellular matrix components in the model rhizobacteria *Pseudomonas fluorescens* F113 and other pseudomonads. *Microorganisms* 8:1740. 10.3390/microorganisms8111740 33171989 PMC7716237

[B8] Blin K. Shaw S. Augustijn H. E. Reitz Z. L. Biermann F. Alanjary M. (2023). antiSMASH 7.0: New and improved predictions for detection, regulation, chemical structures and visualisation. *Nucleic Acids Res.* 51 W46–W50. 10.1093/nar/gkad344 37140036 PMC10320115

[B9] Bouteiller M. Gallique M. Bourigault Y. Kosta A. Hardouin J. Massier S. (2020). Crosstalk between the type VI secretion system and the expression of class IV flagellar genes in the *Pseudomonas fluorescens* MFE01 strain. *Microorganisms* 8:622. 10.3390/microorganisms8050622 32344878 PMC7286023

[B10] Burz S. D. Causevic C. Dal, Co A. Dmitrijeva M. Engel P. (2023). From microbiome composition to functional engineering, one step at a time. *Microbiol. Mol. Biol. Rev.* 87 e63–e23. 10.1128/mmbr.00063-23 37947420 PMC10732080

[B11] Buysens S. Heungens K. Poppe J. Hofte M. (1996). Involvement of pyochelin and pyoverdin in suppression of pythium-induced damping-off of tomato by *Pseudomonas aeruginosa* 7NSK2. *Appl. Environ. Microbiol.* 62 865–871. 10.1128/aem.62.3.865-871.1996 16535275 PMC1388800

[B12] Capella-Gutiérrez S. Silla-Martínez J. M. Gabaldón T. (2009). trimAl: A tool for automated alignment trimming in large-scale phylogenetic analyses. *Bioinformatics* 25 1972–1973. 10.1093/bioinformatics/btp348 19505945 PMC2712344

[B13] Cook S. D. (2019). An historical review of phenylacetic acid. *Plant Cell Physiol.* 60 243–254. 10.1093/pcp/pcz004 30649529

[B14] Costacurta A. Vanderleyden J. (1995). Synthesis of phytohormones by plant-associated bacteria. *Crit. Rev. Microbiol.* 21 1–18. 10.3109/10408419509113531 7576148

[B15] D’aes J. Kieu N. P. Léclère V. Tokarski C. Olorunleke F. E. De Maeyer K. (2014). To settle or to move? The interplay between two classes of cyclic lipopeptides in the biocontrol strain *Pseudomonas* CMR12a. *Environ. Microbiol.* 16 2282–2300. 10.1111/1462-2920.12462 24673852

[B16] De Vrieze M. Gloor R. Massana Codina J. Torriani S. Gindro K. L’Haridon F. (2019). Biocontrol activity of three *Pseudomonas* in a newly assembled collection of *Phytophthora infestans* isolates. *Phytopathology* 109 1555–1565. 10.1094/PHYTO-12-18-0487-R 31041882

[B17] de Werra P. Péchy-Tarr M. Keel C. Maurhofer M. (2009). Role of gluconic acid production in the regulation of biocontrol traits of *Pseudomonas fluorescens* CHA0. *Appl. Environ. Microbiol.* 75 4162–4174. 10.1128/AEM.00295-09 19376896 PMC2698339

[B18] Delorme S. Lemanceau P. Christen R. Corberand T. Meyer J.-M. Gardan L. (2002). *Pseudomonas lini* sp. nov., a novel species from bulk and rhizospheric soils. *Int. J. Syst. Evol. Microbiol.* 52 513–523. 10.1099/00207713-52-2-513 11931164

[B19] Dixon P. (2003). VEGAN, a package of R functions for community ecology. *J. Veg. Sci.* 14 927–930. 10.1111/j.1654-1103.2003.tb02228.x

[B20] Dong X. Rao Z. Wu S. Peng F. Xie Z. Long Y. (2023). *Pseudomonas benzopyrenica* sp. nov., isolated from soil, exhibiting high-efficiency degradation of benzo(a)pyrene. *Int. J. Syst. Evol. Microbiol.* 73:006034. 10.1099/ijsem.0.006034 37725099

[B21] Durán D. Bernal P. Vazquez-Arias D. Blanco-Romero E. Garrido-Sanz D. Redondo-Nieto M. (2021). *Pseudomonas fluorescens* F113 type VI secretion systems mediate bacterial killing and adaption to the rhizosphere microbiome. *Sci. Rep.* 11:5772. 10.1038/s41598-021-85218-1 33707614 PMC7970981

[B22] Eichmann R. Richards L. Schäfer P. (2021). Hormones as go-betweens in plant microbiome assembly. *Plant J.* 105 518–541. 10.1111/tpj.15135 33332645 PMC8629125

[B23] Emms D. M. Kelly S. (2019). OrthoFinder: Phylogenetic orthology inference for comparative genomics. *Genome Biol.* 20:238. 10.1186/s13059-019-1832-y 31727128 PMC6857279

[B24] Frapolli M. Pothier J. F. Défago G. Moënne-Loccoz Y. (2012). Evolutionary history of synthesis pathway genes for phloroglucinol and cyanide antimicrobials in plant-associated fluorescent pseudomonads. *Mol. Phylogenet. Evol.* 63 877–890. 10.1016/j.ympev.2012.02.030 22426436

[B25] Furmanczyk E. M. Lipinski L. Dziembowski A. Sobczak A. (2018b). Genomic and functional characterization of environmental strains of SDS-degrading *Pseudomonas* spp., providing a source of new sulfatases. *Front. Microbiol.* 9:1795. 10.3389/fmicb.2018.01795 30174655 PMC6107682

[B26] Furmanczyk E. M. Kaminski M. A. Lipinski L. Dziembowski A. Sobczak A. (2018a). *Pseudomonas laurylsulfatovorans* sp. nov., sodium dodecyl sulfate degrading bacteria, isolated from the peaty soil of a wastewater treatment plant. *Syst. Appl. Microbiol.* 41 348–354. 10.1016/j.syapm.2018.03.009 29752019

[B27] Garrido-Sanz D. Arrebola E. Martínez-Granero F. García-Méndez S. Muriel C. Blanco-Romero E. (2017). Classification of isolates from the *Pseudomonas fluorescens* complex into phylogenomic groups based in group-specific markers. *Front. Microbiol.* 8:413. 10.3389/fmicb.2017.00413 28360897 PMC5350142

[B28] Garrido-Sanz D. Vesga P. Heiman C. M. Altenried A. Keel C. Vacheron J. (2023b). Relation of pest insect-killing and soilborne pathogen-inhibition abilities to species diversification in environmental *Pseudomonas protegens*. *ISME J.* 17 1369–1381. 10.1038/s41396-023-01451-8 37311938 PMC10432460

[B29] Garrido-Sanz D. Èaušević S. Vacheron J. Heiman C. M. Sentchilo V. van der Meer J. R. (2023a). Changes in structure and assembly of a species-rich soil natural community with contrasting nutrient availability upon establishment of a plant-beneficial *Pseudomonas* in the wheat rhizosphere. *Microbiome* 11:214. 10.1186/s40168-023-01660-5 37770950 PMC10540321

[B30] Garrido-Sanz D. Meier-Kolthoff J. P. Göker M. Martín M. Rivilla R. Redondo-Nieto M. (2016). Genomic and genetic diversity within the *Pseudomonas fluorescens* complex. *PLoS One* 11:e0150183. 10.1371/journal.pone.0150183 26915094 PMC4767706

[B31] Garrido-Sanz D. Redondo-Nieto M. Martin M. Rivilla R. (2021). Comparative genomics of the *Pseudomonas corrugata* subgroup reveals high species diversity and allows the description of *Pseudomonas ogarae* sp. nov. *Microb. Genom.* 7:000593. 10.1099/mgen.0.000593 34184980 PMC8461476

[B32] Gieschler S. Fiedler G. Böhnlein C. Grimmler C. Franz C. M. A. P. Kabisch J. (2021). *Pseudomonas kielensis* sp. nov. and *Pseudomonas* baltica sp. nov., isolated from raw milk in Germany. *Int. J. Syst. Evol. Microbiol.* 71:004717. 10.1099/ijsem.0.004717 33620302

[B33] Girard L. Lood C. Höfte M. Vandamme P. Rokni-Zadeh H. van Noort V. (2021). The ever-expanding *Pseudomonas* genus: Description of 43 new species and partition of the *Pseudomonas putida* group. *Microorganisms* 9:1766. 10.3390/microorganisms9081766 34442845 PMC8401041

[B34] Glick B. R. (2005). Modulation of plant ethylene levels by the bacterial enzyme ACC deaminase. *FEMS Microbiol. Lett.* 251 1–7. 10.1016/j.femsle.2005.07.030 16099604

[B35] Glick B. R. Todorovic B. Czarny J. Cheng Z. Duan J. McConkey B. (2007). Promotion of plant growth by bacterial ACC deaminase. *Crit. Rev. Plant Sci.* 26 227–242. 10.1080/07352680701572966

[B36] Gordee R. S. Matthews T. R. (1969). Systemic antifungal activity of pyrrolnitrin. *Appl. Microbiol.* 17 690–694. 10.1128/am.17.5.690-694.1969 5785951 PMC377781

[B37] Goris J. Konstantinidis K. T. Klappenbach J. A. Coenye T. Vandamme P. Tiedje J. M. (2007). DNA–DNA hybridization values and their relationship to whole-genome sequence similarities. *Int. J. Syst. Evol. Microbiol.* 57 81–91. 10.1099/ijs.0.64483-0 17220447

[B38] Grosse C. Brandt N. Van Antwerpen P. Wintjens R. Matthijs S. (2023). Two new siderophores produced by *Pseudomonas* sp. NCIMB 10586: The anti-oomycete non-ribosomal peptide synthetase-dependent mupirochelin and the NRPS-independent triabactin. *Front. Microbiol.* 14:1143861. 10.3389/fmicb.2023.1143861 37032897 PMC10080011

[B39] Gu Z. Eils R. Schlesner M. (2016). Complex heatmaps reveal patterns and correlations in multidimensional genomic data. *Bioinformatics* 32 2847–2849. 10.1093/bioinformatics/btw313 27207943

[B40] Haas D. Défago G. (2005). Biological control of soil-borne pathogens by fluorescent pseudomonads. *Nat. Rev. Microbiol.* 3 307–319. 10.1038/nrmicro1129 15759041

[B41] Haft D. H. Selengut J. D. Richter R. A. Harkins D. Basu M. K. Beck E. (2013). TIGRFAMs and genome properties in 2013. *Nucleic Acids Res.* 41 D387–D395. 10.1093/nar/gks1234 23197656 PMC3531188

[B42] Harmsen N. Vesga P. Glauser G. Klötzli F. Heiman C. M. Altenried A. (2024). Natural soil suppressiveness against soilborne phytopathogens extends to the control of insect pest. *Microbiome (in press)*. 10.1101/2024.03.12.584584 bioRxiv:2024.03.12.584584. PMC11251354 39014485

[B43] Haskett T. L. Tkacz A. Poole P. S. (2021). Engineering rhizobacteria for sustainable agriculture. *ISME J.* 15 949–964. 10.1038/s41396-020-00835-4 33230265 PMC8114929

[B44] Hauser E. Kämpfer P. Busse H.-J. (2004). *Pseudomonas psychrotolerans* sp. nov. *Int. J. Syst. Evol. Microbiol.* 54 1633–1637. 10.1099/ijs.0.03024-0 15388721

[B45] Hauth F. Buck H. Hartig J. S. (2022). *Pseudomonas canavaninivorans* sp. nov., isolated from bean rhizosphere. *Int. J. Syst. Evol. Microbiol.* 72:005203. 10.1099/ijsem.0.005203 35072599

[B46] Hayat R. Ali S. Amara U. Khalid R. Ahmed I. (2010). Soil beneficial bacteria and their role in plant growth promotion: A review. *Ann. Microbiol.* 60 579–598. 10.1007/s13213-010-0117-1

[B47] Ho Y.-N. Hoo S. Y. Wang B.-W. Hsieh C.-T. Lin C.-C. Sun C.-H. (2021). Specific inactivation of an antifungal bacterial siderophore by a fungal plant pathogen. *ISME J.* 15 1858–1861. 10.1038/s41396-020-00871-0 33619352 PMC8163733

[B48] Howell R. C. Stipanovic R. D. (1980). Suppression of *Pythium ultimum*-induced damping-off of cotton seedlings by *Pseudomonas fluorescens* and its antibiotic, pyoluteorin. *Phytopathology* 70 712–715. 10.1094/Phyto-70-712

[B49] Huerta-Cepas J. Forslund K. Coelho L. P. Szklarczyk D. Jensen L. J. von Mering C. (2017). fast genome-wide functional annotation through orthology assignment by eggNOG-Mapper. *Mol. Biol. Evol.* 34 2115–2122. 10.1093/molbev/msx148 28460117 PMC5850834

[B50] Huerta-Cepas J. Szklarczyk D. Heller D. Hernández-Plaza A. Forslund S. K. Cook H. (2019). eggNOG 5.0: A hierarchical, functionally and phylogenetically annotated orthology resource based on 5090 organisms and 2502 viruses. *Nucleic Acids Res.* 47 D309–D314. 10.1093/nar/gky1085 30418610 PMC6324079

[B51] Iavicoli A. Boutet E. Buchala A. Métraux J.-P. (2003). Induced systemic resistance in *Arabidopsis thaliana* in response to root inoculation with *Pseudomonas fluorescens* CHA0. *Mol. Plant. Microbe Interact.* 16 851–858. 10.1094/MPMI.2003.16.10.851 14558686

[B52] Ilangumaran G. Smith D. L. (2017). Plant growth promoting rhizobacteria in amelioration of salinity stress: A systems biology perspective. *Front. Plant Sci.* 8:1768. 10.3389/fpls.2017.01768 29109733 PMC5660262

[B53] Jahanshah G. Yan Q. Gerhardt H. Pataj Z. Lämmerhofer M. Pianet I. (2019). Discovery of the cyclic lipopeptide gacamide A by genome mining and repair of the defective GacA regulator in *Pseudomonas fluorescens* Pf0-1. *J. Nat. Prod.* 82 301–308. 10.1021/acs.jnatprod.8b00747 30666877

[B54] Jain C. Rodriguez-R L. M. Phillippy A. M. Konstantinidis K. T. Aluru S. (2018). High throughput ANI analysis of 90K prokaryotic genomes reveals clear species boundaries. *Nat. Commun.* 9:5114. 10.1038/s41467-018-07641-9 30504855 PMC6269478

[B55] Kaplan A. R. Musaev D. G. Wuest W. M. (2021). Pyochelin biosynthetic metabolites bind iron and promote growth in pseudomonads demonstrating siderophore-like activity. *ACS Infect. Dis.* 7 544–551. 10.1021/acsinfecdis.0c00897 33577297 PMC8322966

[B56] Katoh K. Standley D. M. (2013). MAFFT multiple sequence alignment software version 7: Improvements in Performance and Usability. *Mol. Biol. Evol.* 30 772–780. 10.1093/molbev/mst010 23329690 PMC3603318

[B57] Kawasaki S. Arai H. Kodama T. Igarashi Y. (1997). Gene cluster for dissimilatory nitrite reductase (nir) from *Pseudomonas aeruginosa*: Sequencing and identification of a locus for heme d1 biosynthesis. *J. Bacteriol.* 179 235–242. 10.1128/jb.179.1.235-242.1997 8982003 PMC178684

[B58] Keel C. (2016). A look into the toolbox of multi-talents: Insect pathogenicity determinants of plant-beneficial pseudomonads. *Environ. Microbiol.* 18 3207–3209. 10.1111/1462-2920.13462 27450048

[B59] Keel C. Schnider U. Maurhofer M. Voisard C. Laville J. Burger U. (1992). Suppression of root diseases by *Pseudomonas fluorescens* CHA0: Importance of the bacterial secondary metabolite 2, 4-diacetylphloroglucinol. *Mol. Plant. Microbe Interact.* 5 4–13.

[B60] Kim Y. Cho J.-Y. Kuk J.-H. Moon J.-H. Cho J.-I. Kim Y.-C. (2004). Identification and antimicrobial activity of phenylacetic acid produced by *Bacillus licheniformis* isolated from fermented soybean, Chungkook-Jang. *Curr. Microbiol.* 48 312–317. 10.1007/s00284-003-4193-3 15057459

[B61] Kodama K. Kimura N. Komagata K. (1985). Two new species of *Pseudomonas*: *P. oryzihabitans* isolated from rice paddy and clinical specimens and *P. luteola* isolated from clinical specimens. *Int. J. Syst. Evol. Microbiol.* 35 467–474. 10.1099/00207713-35-4-467

[B62] Lalucat J. Gomila M. Mulet M. Zaruma A. García-Valdés E. (2022). Past, present and future of the boundaries of the *Pseudomonas* genus: Proposal of *Stutzerimonas* gen. nov. *Syst. Appl. Microbiol.* 45:126289. 10.1016/j.syapm.2021.126289 34920232

[B63] Lalucat J. Mulet M. Gomila M. García-Valdés E. (2020). Genomics in bacterial taxonomy: Impact on the genus *Pseudomonas*. *Genes* 11:139. 10.3390/genes11020139 32013079 PMC7074058

[B64] Laville J. Blumer C. Von Schroetter C. Gaia V. Défago G. Keel C. (1998). Characterization of the *hcnABC* gene cluster encoding hydrogen cyanide synthase and anaerobic regulation by ANR in the strictly aerobic biocontrol agent *Pseudomonas fluorescens* CHA0. *J. Bacteriol.* 180 3187–3196. 10.1128/jb.180.12.3187-3196.1998 9620970 PMC107821

[B65] Lind P. A. Farr A. D. Rainey P. B. (2017). Evolutionary convergence in experimental *Pseudomonas* populations. *ISME J.* 11 589–600. 10.1038/ismej.2016.157 27911438 PMC5322309

[B66] Loper J. E. Hassan K. A. Mavrodi D. V. Davis E. W. II Lim C. K. Shaffer B. T. (2012). Comparative genomics of plant-associated *Pseudomonas* spp.: Insights into diversity and inheritance of traits involved in multitrophic interactions. *PLoS Genet.* 8:e1002784. 10.1371/journal.pgen.1002784 22792073 PMC3390384

[B67] Loper J. E. Henkels M. D. Rangel L. I. Olcott M. H. Walker F. L. Bond K. L. (2016). Rhizoxin analogs, orfamide A and chitinase production contribute to the toxicity of *Pseudomonas protegens* strain Pf-5 to Drosophila melanogaster. *Environ. Microbiol.* 18 3509–3521. 10.1111/1462-2920.13369 27130686

[B68] Lopes L. D. Davis E. W. Pereira Silva M. C. Weisberg A. J. Bresciani L. Chang J. H. (2018). Tropical soils are a reservoir for fluorescent *Pseudomonas* spp. biodiversity. *Environ. Microbiol.* 20 62–74. 10.1111/1462-2920.13957 29027341

[B69] Marchand P. A. Weller D. M. Bonsall R. F. (2000). Convenient synthesis of 2,4-diacetylphloroglucinol, a natural antibiotic involved in the control of take-all disease of wheat. *J. Agric. Food Chem.* 48 1882–1887. 10.1021/jf9907135 10820109

[B70] Mauch-Mani B. Baccelli I. Luna E. Flors V. (2017). Defense priming: An adaptive part of induced resistance. *Annu. Rev. Plant Biol.* 68 485–512. 10.1146/annurev-arplant-042916-041132 28226238

[B71] Meier-Kolthoff J. P. Auch A. F. Klenk H.-P. Göker M. (2013). Genome sequence-based species delimitation with confidence intervals and improved distance functions. *BMC Bioinform.* 14:60. 10.1186/1471-2105-14-60 23432962 PMC3665452

[B72] Meier-Kolthoff J. P. Carbasse J. S. Peinado-Olarte R. L. Göker M. (2022). TYGS and LPSN: A database tandem for fast and reliable genome-based classification and nomenclature of prokaryotes. *Nucleic Acids Res.* 50 D801–D807. 10.1093/nar/gkab902 34634793 PMC8728197

[B73] Mercado-Blanco J. van der Drift K. M. G. M. Olsson P. E. Thomas-Oates J. E. van Loon L. C. Bakker P. A. H. M. (2001). Analysis of the *pmsCEAB* gene cluster involved in biosynthesis of salicylic acid and the siderophore pseudomonine in the biocontrol strain *Pseudomonas fluorescens* WCS374. *J. Bacteriol.* 183 1909–1920. 10.1128/jb.183.6.1909-1920.2001 11222588 PMC95085

[B74] Minh B. Q. Schmidt H. A. Chernomor O. Schrempf D. Woodhams M. D. von Haeseler A. (2020). IQ-TREE 2: New models and efficient methods for phylogenetic inference in the genomic era. *Mol. Biol. Evol.* 37 1530–1534. 10.1093/molbev/msaa015 32011700 PMC7182206

[B75] Mistry J. Chuguransky S. Williams L. Qureshi M. Salazar G. A. Sonnhammer E. L. L. (2021). Pfam: The protein families database in 2021. *Nucleic Acids Res.* 49 D412–D419. 10.1093/nar/gkaa913 33125078 PMC7779014

[B76] Mulet M. Gomila M. Busquets A. Sánchez D. Lalucat J. García-Valdés E. (2024). Genome-based taxonomy of species in the *Pseudomonas syringae* and *Pseudomonas lutea* phylogenetic groups and proposal of *Pseudomonas maioricensis* sp. nov., isolated from agricultural soil. *Microorganisms* 12:460. 10.3390/microorganisms12030460 38543511 PMC10974278

[B77] Naseem H. Ahsan M. Shahid M. A. Khan N. (2018). Exopolysaccharides producing rhizobacteria and their role in plant growth and drought tolerance. *J. Basic Microbiol.* 58 1009–1022. 10.1002/jobm.201800309 30183106

[B78] Olanrewaju O. S. Glick B. R. Babalola O. O. (2017). Mechanisms of action of plant growth promoting bacteria. *World J. Microbiol. Biotechnol.* 33:197. 10.1007/s11274-017-2364-9 28986676 PMC5686270

[B79] Oni F. E. Olorunleke O. F. Höfte M. (2019). Phenazines and cyclic lipopeptides produced by *Pseudomonas* sp. CMR12a are involved in the biological control of *Pythium myriotylum* on cocoyam (*Xanthosoma sagittifolium*). *Biol. Control* 129 109–114. 10.1016/j.biocontrol.2018.10.005

[B80] Parks D. H. Imelfort M. Skennerton C. T. Hugenholtz P. Tyson G. W. (2015). CheckM: Assessing the quality of microbial genomes recovered from isolates, single cells, and metagenomes. *Genome Res.* 25 1043–1055. 10.1101/gr.186072.114 25977477 PMC4484387

[B81] Patten C. L. Glick B. R. (1996). Bacterial biosynthesis of indole-3-acetic acid. *Can. J. Microbiol.* 42 207–220. 10.1139/m96-032 8868227

[B82] Péchy-Tarr M. Bruck D. J. Maurhofer M. Fischer E. Vogne C. Henkels M. D. (2008). Molecular analysis of a novel gene cluster encoding an insect toxin in plant-associated strains of *Pseudomonas fluorescens*. *Environ. Microbiol.* 10 2368–2386. 10.1111/j.1462-2920.2008.01662.x 18484997

[B83] Pentz J. T. Lind P. A. (2021). Forecasting of phenotypic and genetic outcomes of experimental evolution in *Pseudomonas* protegens. *PLoS Genet.* 17:e1009722. 10.1371/journal.pgen.1009722 34351900 PMC8370652

[B84] Prakash O. Verma M. Sharma P. Kumar M. Kumari K. Singh A. (2007). Polyphasic approach of bacterial classification rtf54– An overview of recent advances. *Indian J. Microbiol.* 47 98–108. 10.1007/s12088-007-0022-x 23100651 PMC3450112

[B85] Qin S. Xiao W. Zhou C. Pu Q. Deng X. Lan L. (2022). *Pseudomonas aeruginosa*: Pathogenesis, virulence factors, antibiotic resistance, interaction with host, technology advances and emerging therapeutics. *Signal Transduct. Target. Ther.* 7:199. 10.1038/s41392-022-01056-1 35752612 PMC9233671

[B86] Ramasamy D. Mishra A. K. Lagier J.-C. Padhmanabhan R. Rossi M. Sentausa E. (2014). A polyphasic strategy incorporating genomic data for the taxonomic description of novel bacterial species. *Int. J. Syst. Evol. Microbiol.* 64 384–391. 10.1099/ijs.0.057091-0 24505076

[B87] Ramette A. Frapolli M. Saux M. F.-L. Gruffaz C. Meyer J.-M. Défago G. (2011). *Pseudomonas protegens* sp. nov., widespread plant-protecting bacteria producing the biocontrol compounds 2,4-diacetylphloroglucinol and pyoluteorin. *Syst. Appl. Microbiol.* 34 180–188. 10.1016/j.syapm.2010.10.005 21392918

[B88] Ramette A. Moënne-Loccoz Y. Défago G. (2006). Genetic diversity and biocontrol potential of fluorescent pseudomonads producing phloroglucinols and hydrogen cyanide from Swiss soils naturally suppressive or conducive to *Thielaviopsis basicola*-mediated black root rot of tobacco. *FEMS Microbiol. Ecol.* 55 369–381. 10.1111/j.1574-6941.2005.00052.x 16466376

[B89] Richardson D. J. Berks B. C. Russell D. A. Spiro S. Taylor C. J. (2001). Functional, biochemical and genetic diversity of prokaryotic nitrate reductases. *Cell. Mol. Life Sci. CMLS* 58 165–178. 10.1007/PL00000845 11289299 PMC11146511

[B90] Richter M. Rosselló-Móra R. (2009). Shifting the genomic gold standard for the prokaryotic species definition. *Proc. Natl. Acad. Sci. U.S.A.* 106 19126–19131. 10.1073/pnas.0906412106 19855009 PMC2776425

[B91] Schneider C. A. Rasband W. S. Eliceiri K. W. (2012). NIH Image to ImageJ: 25 years of image analysis. *Nat. Methods* 9 671–675. 10.1038/nmeth.2089 22930834 PMC5554542

[B92] Seemann T. (2014). Prokka: Rapid prokaryotic genome annotation. *Bioinformatics* 30 2068–2069. 10.1093/bioinformatics/btu153 24642063

[B93] Silby M. W. Cerdeño-Tárraga A. M. Vernikos G. S. Giddens S. R. Jackson R. W. Preston G. M. (2009). Genomic and genetic analyses of diversity and plant interactions of *Pseudomonas fluorescens*. *Genome Biol.* 10:R51. 10.1186/gb-2009-10-5-r51 19432983 PMC2718517

[B94] Silby M. W. Winstanley C. Godfrey S. A. C. Levy S. B. Jackson R. W. (2011). *Pseudomonas* genomes: Diverse and adaptable. *FEMS Microbiol. Rev.* 35 652–680. 10.1111/j.1574-6976.2011.00269.x 21361996

[B95] Soutourina O. A. Bertin P. N. (2003). Regulation cascade of flagellar expression in Gram-negative bacteria. *FEMS Microbiol. Rev.* 27 505–523. 10.1016/S0168-6445(03)00064-0 14550943

[B96] Spaepen S. Vanderleyden J. Remans R. (2007). Indole-3-acetic acid in microbial and microorganism-plant signaling. *FEMS Microbiol. Rev.* 31 425–448. 10.1111/j.1574-6976.2007.00072.x 17509086

[B97] Spiers A. J. Buckling A. Rainey P. B. (2000). The causes of *Pseudomonas* diversity. *Microbiology* 146 2345–2350. 10.1099/00221287-146-10-2345 11021911

[B98] Stow P. R. Reitz Z. L. Johnstone T. C. Butler A. (2021). Genomics-driven discovery of chiral triscatechol siderophores with enantiomeric Fe(iii) coordination. *Chem. Sci.* 12 12485–12493. 10.1039/D1SC03541J 34603680 PMC8480324

[B99] Terlouw B. R. Blin K. Navarro-Muñoz J. C. Avalon N. E. Chevrette M. G. Egbert S. (2023). MIBiG 3.0: A community-driven effort to annotate experimentally validated biosynthetic gene clusters. *Nucleic Acids Res.* 51 D603–D610. 10.1093/nar/gkac1049 36399496 PMC9825592

[B100] Teufel R. Mascaraque V. Ismail W. Voss M. Perera J. Eisenreich W. (2010). Bacterial phenylalanine and phenylacetate catabolic pathway revealed. *Proc. Natl. Acad. Sci. U.S.A.* 107 14390–14395. 10.1073/pnas.1005399107 20660314 PMC2922514

[B101] Todai T. Takahashi F. Yasuoka S. Sato T. Abe K. Takikawa Y. (2022). *Pseudomonas amygdali* (syn. *Pseudomonas savastanoi*) pv. adzukicola pv. nov., causal agent of bacterial stem rot of adzuki bean. *J. Gen. Plant Pathol.* 88 358–371. 10.1007/s10327-022-01084-3

[B102] Trivedi P. Leach J. E. Tringe S. G. Sa T. Singh B. K. (2020). Plant–microbiome interactions: From community assembly to plant health. *Nat. Rev. Microbiol.* 18 607–621. 10.1038/s41579-020-0412-1 32788714

[B103] Turnbull G. A. Morgan J. A. W. Whipps J. M. Saunders J. R. (2001). The role of bacterial motility in the survival and spread of *Pseudomonas fluorescens* in soil and in the attachment and colonisation of wheat roots. *FEMS Microbiol. Ecol.* 36 21–31. 10.1111/j.1574-6941.2001.tb00822.x 11377770

[B104] Vacheron J. Desbrosses G. Renoud S. Padilla R. Walker V. Muller D. (2018). Differential contribution of plant-beneficial functions from *Pseudomonas kilonensis* F113 to root system architecture alterations in *Arabidopsis thaliana* and *Zea mays*. *Mol. Plant. Microbe Interact.* 31 212–223. 10.1094/MPMI-07-17-0185-R 28971723

[B105] Vacheron J. Heiman C. M. Garneau J. R. Kupferschmied P. de Jonge R. Garrido-Sanz D. (2023). Molecular and evolutionary basis of O-antigenic polysaccharide-driven phage sensitivity in environmental pseudomonads. *Microbiol. Spectr.* 11 e2049–e2023. 10.1128/spectrum.02049-23 37800913 PMC10715155

[B106] Vacheron J. Péchy-Tarr M. Brochet S. Heiman C. M. Stojiljkovic M. Maurhofer M. (2019). T6SS contributes to gut microbiome invasion and killing of an herbivorous pest insect by plant-beneficial *Pseudomonas protegens*. *ISME J.* 13 1318–1329. 10.1038/s41396-019-0353-8 30683920 PMC6474223

[B107] Vandamme P. Pot B. Gillis M. Kersters K. Swings J. (1996). Polyphasic taxonomy, a consensus approach to bacterial systematics. *Microbiol. Rev.* 60 407–438. 10.1128/mr.60.2.407-438.1996 8801440 PMC239450

[B108] Verhille S. Baida N. Dabboussi F. Izard D. Leclerc H. (1999). Taxonomic study of bacteria isolated from natural mineral waters: Proposal of *Pseudomonas jessenii* sp. nov. and *Pseudomonas mandelii* sp. nov. *Syst. Appl. Microbiol.* 22 45–58. 10.1016/S0723-2020(99)80027-7 10188278

[B109] Voisard C. Keel C. Haas D. Défago G. (1989). Cyanide production by *Pseudomonas fluorescens* helps suppress black root rot of tobacco under gnotobiotic conditions. *EMBO J.* 8 351–358. 10.1002/j.1460-2075.1989.tb03384.x 16453871 PMC400813

[B110] Wang X. He S. Guo H.-B. Thin K. K. Gao J. Wang Y. (2020). *Pseudomonas rhizoryzae* sp. nov., isolated from rice. *Int. J. Syst. Evol. Microbiol.* 70 944–950. 10.1099/ijsem.0.003852 31751195

[B111] Weller D. M. Mavrodi D. V. van Pelt J. A. Pieterse C. M. J. van Loon L. C. Bakker P. A. H. M. (2012). Induced systemic resistance in *Arabidopsis thaliana* against *Pseudomonas syringae* pv. tomato by 2,4-diacetylphloroglucinol-producing *Pseudomonas fluorescens*. *Phytopathology* 102 403–412. 10.1094/PHYTO-08-11-0222 22409433

[B112] Xin X.-F. Kvitko B. He S. Y. (2018). *Pseudomonas syringae*: What it takes to be a pathogen. *Nat. Rev. Microbiol.* 16 316–328. 10.1038/nrmicro.2018.17 29479077 PMC5972017

[B113] Zumft W. G. (1997). Cell biology and molecular basis of denitrification. *Microbiol. Mol. Biol. Rev.* 61 533–616. 10.1128/mmbr.61.4.533-616.1997 9409151 PMC232623

