# Supplementary material for: Expanding the Pseudomonas diversity of the wheat rhizosphere: four novel species antagonizing fungal phytopathogens and with plant-beneficial properties
Source: Front Microbiol. 2024 Jul 15;15:1440341. doi: 10.3389/fmicb.2024.1440341 (PMC11284033; doi:10.3389/fmicb.2024.1440341)
Supplement: Supplementary file 2 [file Image_1.PDF]

Supplementary Figures

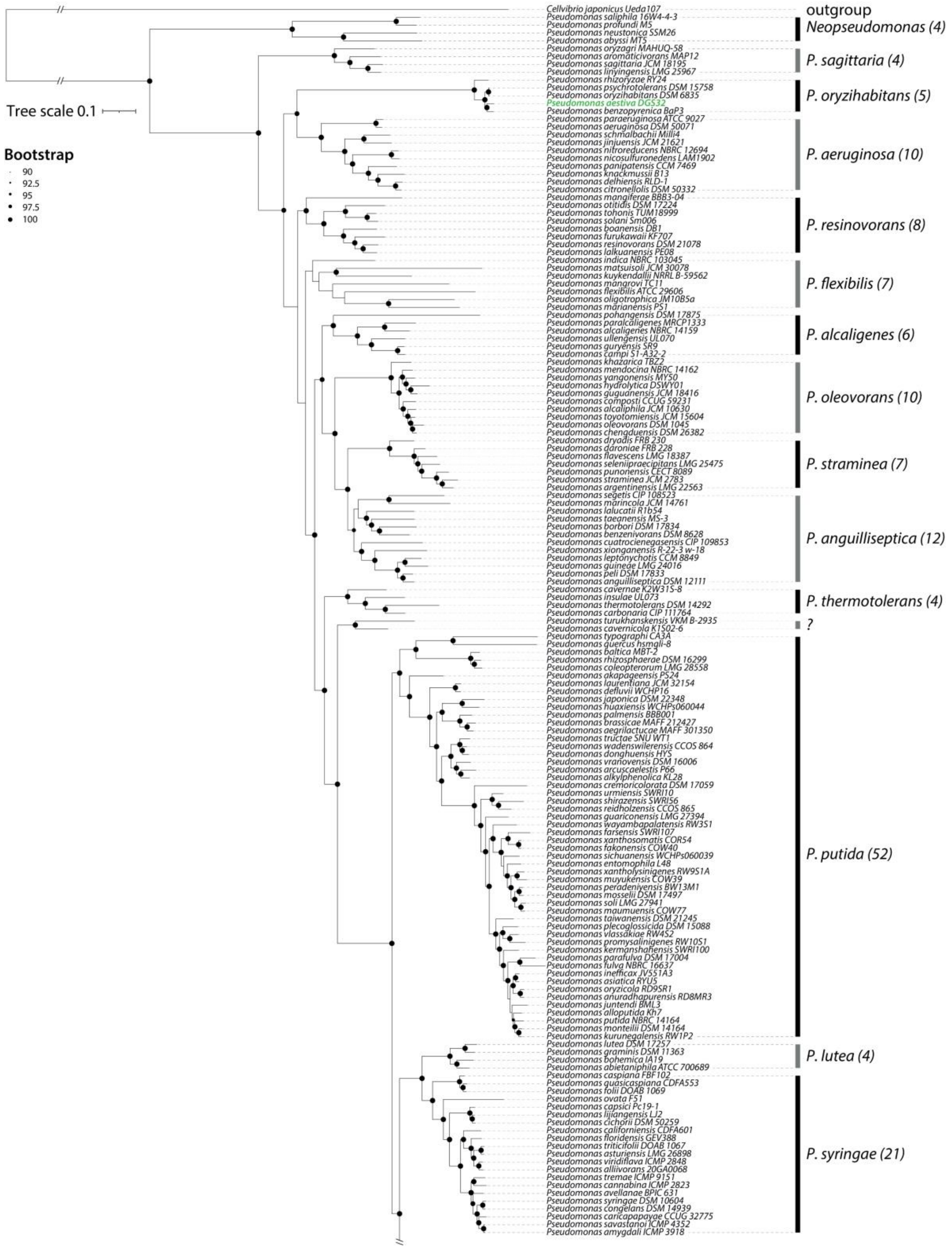

Supplementary Figure S1. Phylogeny of the *Pseudomonas* genus. Maximum-likelihood phylogeny based on the concatenated sequences of 912 single-copy protein sequences of type strains. *Cellvibrio japonicus* Ueda107 was used as the outgroup. Major phylogenetic groups are indicated at the right, with gray/black lines and number of species under brackets. Subgroups belonging to the *P. fluorescens* species complex are indicated in light/dark green. The four strains characterized in this study are highlighted in green. Unnamed groups are indicated by a question mark. Continues on the next page.

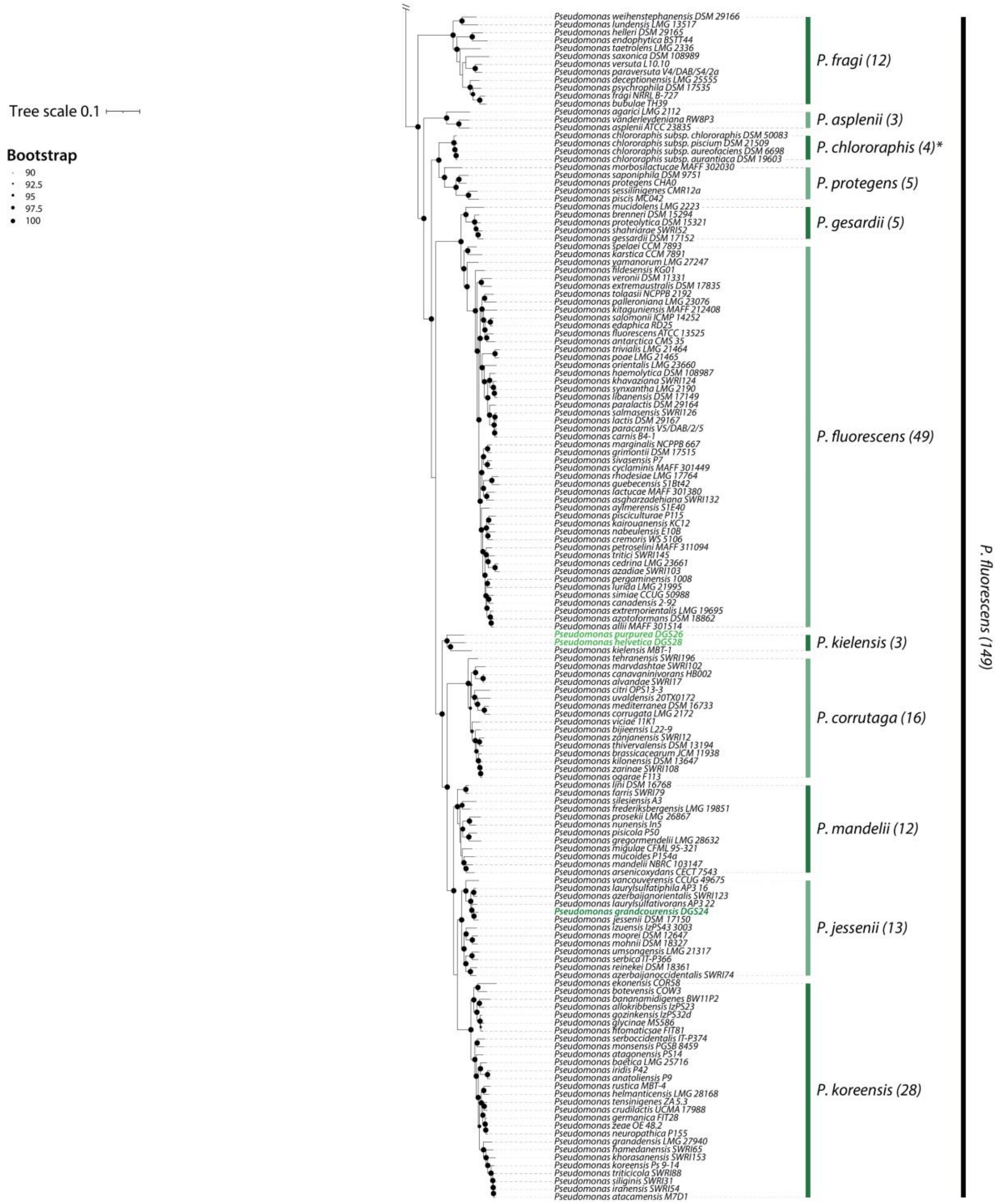

Supplementary Figure S1. Continued.
